# Increased Immune-Regulatory Receptor Expression on Effector T Cells as Early Indicators of Relapse Following Autologous Stem Cell Transplantation for Multiple Myeloma

**DOI:** 10.3389/fimmu.2021.618610

**Published:** 2021-02-25

**Authors:** Lydia Lee, Nouf Alrasheed, Garima Khandelwal, Evelyn Fitzsimons, Huw Richards, William Wilson, Selina J. Chavda, Jake Henry, Lucia Conde, Marc Robert De Massy, Melody Chin, Daria Galas-Filipowicz, Javier Herrero, Benny Chain, Sergio A. Quezada, Kwee Yong

**Affiliations:** ^1^ Research Department of Hematology, Cancer Institute, University College London, London, United Kingdom; ^2^ Bill Lyons Informatics Centre, Cancer Institute, University College London, London, United Kingdom; ^3^ Cancer Research UK & UCL Cancer Trials Centre, London, United Kingdom; ^4^ Cancer Immunology Unit, Research Department of Hematology, University College London Cancer Institute, London, United Kingdom; ^5^ Department of Immunology, University College London, London, United Kingdom

**Keywords:** multiple myeloma, immune phenotype, PD-1, Ki-67, T cell receptor, autologous stem cell transplant

## Abstract

The benefit of autologous stem cell transplantation (ASCT) in newly diagnosed myeloma patients, apart from supporting high dose chemotherapy, may include effects on T cell function in the bone marrow (BM). We report our exploratory findings on marrow infiltrating T cells early post-ASCT (day+100), examining phenotype and T cell receptor (TCR) repertoire, seeking correlations with timing of relapse. Compared to healthy donors (HD), we observed an increase in regulatory T cells (CD4+FoxP3+, Tregs) with reduction in CD4 T cells, leading to lower CD4:8 ratios. Compared to paired pre-treatment marrow, both CD4 and CD8 compartments showed a reduction in naïve, and increase in effector memory subsets, suggestive of a more differentiated phenotype. This was supported by increased levels of several immune-regulatory and activation proteins (ICOS, PD-1, LAG-3, CTLA-4 and GzmB) when compared with HD. Unsupervised analysis identified a patient subgroup with shorter PFS (p=0.031) whose BM contained increased Tregs, and higher immune-regulatory markers (ICOS, PD-1, LAG-3) on effector T cells. Using single feature analysis, higher frequencies of marrow PD-1+ on CD4+FoxP3- cells and Ki67+ on CD8 cells were independently associated with early relapse. Finally, studying paired pre-treatment and post-ASCT BM (n=5), we note reduced abundance of TCR sequences at day+100, with a greater proportion of expanded sequences indicating a more focused persistent TCR repertoire. Our findings indicate that, following induction chemotherapy and ASCT, marrow T cells demonstrate increased activation and differentiation, with TCR repertoire focusing. Pending confirmation in larger series, higher levels of immune-regulatory proteins on T cell effectors at day+100 may indicate early relapse.

## Introduction

Current standard of care in young fit patients with newly diagnosed multiple myeloma (MM) is several cycles of induction chemotherapy followed by high dose melphalan and autologous stem cell transplantation (ASCT) (1–3). While high dose therapy eradicates residual tumor cells, an increasing body of evidence suggests that immunological mechanisms may also contribute to the prolongation of disease-free survival following ASCT. In a murine myeloma transplant model, T cell dependent myeloma control was an essential mediator of disease remission, and the priming of naïve T cells to tumor was observed (4). The expansion of T cell clones post-ASCT and early lymphocyte recovery has been associated with longer PFS, suggesting that early immune reconstitution is important in controlling disease progression in MM patients (5–8). Furthermore, MM patients suffering early relapse post-ASCT were observed to have an increased frequency of exhausted or senescent T cells (9). Hence, the immune “fitness” of the reconstituting marrow cells may influence clinical outcomes in patients receiving ASCT.

We examined the T cell phenotype of marrow infiltrating lymphocytes in patients at day 100 (D100) post-ASCT, exploring associations with disease free survival. We observed the presence of activated CD4 and CD8 cells, but also significant expression of markers of immune regulation, activation, and proliferation and increased frequency of T-regulatory cells (Treg) when compared to healthy controls. We also examined the T cell receptor (TCR) repertoire of patients post-transplant, seeking key changes compared to diagnostic marrow samples that might provide clues regarding reactivity.

## Methods

### Patients and Controls

Two cohorts of patients were studied. Firstly, bone marrow (BM) aspirates were obtained from 61 MM patients at D100 post-ASCT (**Supplementary Table 1**). A second group of nine patients were studied, from whom we had paired diagnostic (pre-treatment) and D100 post-ASCT samples **(** **Supplementary Table 2**). Written informed consent was obtained from all patients (Research ethics committee reference: 07/Q0502/17). Control BM aspirates were collected from healthy volunteers undergoing BM harvesting with Anthony Nolan, and subjects undergoing BM sampling who had no known hematological diagnosis (**Supplementary Table 1**)(REC reference: 15/YH/0311). All BM samples were collected in ethylenediamine-tetraacetic acid (EDTA) and processed within 24 h. Patients were considered to have adverse risk disease cytogenetics if fluorescent-in-situ-hybridization studies demonstrated one of: t(4,14), t(14,16), t(14,20), and del(17p). Progression free survival (PFS) was defined as time from ASCT to first progression or death and response criteria was as per International Myeloma Working Group criteria (10).

### Isolation of Mononuclear Cells from BM Aspirates

BM mononuclear cells (MNCs) were isolated by Ficoll Paque (GE Healthcare) centrifugation and cryopreserved in fetal bovine serum (FBS) (Gibco) containing 10% DMSO (Sigma Aldrich). Aliquots of cells following depletion of CD138 positive cells (i.e., CD138- fraction, MACS beads, Miltenyi) were subsequently thawed for antibody staining and flow cytometry or T cell receptor (TCR) sequencing

### Flow Cytometry Analysis

Antigen staining was performed using the antibodies outlined in **Supplementary Table 3**. For the intracellular antigen staining, cells were fixed/permeabilized using the FoxP3 Transcription Factor Staining Buffer Set (eBioscience) according to manufacturer’s protocol. Acquisition by BD LSR II Fortessa or BD Symphony (BD Biosciences). Expression of markers is expressed as proportion of parent cell subset, ie. CD4+PD-1+ refers to PD-1+ cells as percentage of CD4+ effectors, unless stated otherwise.

Unsupervised clustering was performed on the basis of the percentage of cells positive for that particular antigen within the relevant parent population (Treg, CD4eff, or CD8 T cells, see above) using the glmnet R package (https://cran.r-project.org/web/packages/glmnet/index.html). The analysis produced unsupervised hierarchical clusters of patients and heatmap of the scaled T cell subsets expressing markers of immune regulation, activation and proliferation. Flow cytometric data were analyzed with FlowJo version 10 (Tree Star Inc), Dimensionality reduction was achieved by scaling down to 10,000 events, UMAP and clustering by phenograph accessed by FlowJo Plugins.

### TCR-Sequencing and Computational Analysis

TCR-Sequencing was performed on 5 paired patient samples at baseline (BL) and post-ASCT according to the published protocol (11). Briefly, RNA was extracted from CD138 depleted BM MNCs (ReliaPrep™ RNA Miniprep System, Promega), quantified and integrity assessed (Qubit, ThermoFisher). Library preparation then consisted of DNase treatment, reverse transcription of TCR RNA, ligation, purification and amplification steps, and incorporation of sequencing primers and indices. The samples were then assessed using TapeStation (Agilent) and sequenced on Illumina NextSeq. Raw sequencing data was processed using Decombinator version 4 (12) for TCR identification, error correction and CDR3 extraction (https://github.com/innate2adaptive/Decombinator). As the number of reads were consistently higher for the BL samples as compared to ASCT samples we controlled for the difference in sample size by performing statistical comparisons and clustering analyses after BL samples were iteratively sub-sampled 100 times to obtain equal number of TCR reads to their paired post-ASCT sample.

Renyi Entropy was calculated using vegan package (https://cran.r-project.org/web/packages/vegan/index.html) in R. Each sample was sub-sampled 100 times as indicated above (3000 TCR for α-chain and 9000 TCR for β-chain).

TCR sequences exceeding a frequency of 1/1,000 were termed expanded TCRs (13). The top 50 expanded CDR3 sequences from each sample were used for clustering. The cluster networks were defined using an amino acid triplet string kernel as previously described (13, 14). The threshold for similarity was 0.80, a level which resulted in minimal clustering of control unexpanded TCRs.

### Statistical Analysis

The percentage of any given marker is designated as “frequency” (of that marker) within the relevant Treg (CD4+FoxP3+), CD4eff(CD4+FoxP3-), or CD8 populations. Statistical analyses were performed with GraphPad Prism software (Prism 7). P values were calculated using statistical tests as indicated in text. PFS was estimated using Kaplan-Meier methods with log-rank test. A multivariate Cox regression model was used to evaluate the independent contribution of variables. All tests of significance were 2-sided and p values ≤0.05 considered statistically significant

## Results

### Clinical Characteristics of Patient Cohort

In the first cohort of 61 patients with BM samples studied at 3 months post-ASCT (D100), 54 received their first ASCT, while the other seven received a second salvage ASCT at relapse. All except two patients had received proteasome inhibitor based induction therapy. Of 50 patients for whom genetic information was available, 10 had adverse risk genetics. All were conditioned with high dose melphalan and 13(21.3%) received consolidation/maintenance, starting after day 100. Patient characteristics are detailed in **Supplementary Table 1**. At a median follow up of 17 months (range 3–54), the median PFS was 24 months. At time of BM sampling (D100), 24.6% had achieved complete response (CR), 47.5% very good partial response (VGPR), 21.3% partial response (PR), 4.9% stable disease (SD), and none had progressive disease (PD). Deeper response (CR/VGPR) was associated with longer PFS (p=0.003), as was ISS stage I vs II/III (p=0.012). There was a trend for longer PFS with standard risk genetics (standard risk vs high risk, p=0.088), and a trend for a benefit with consolidation/maintenance (p=0.11) (**Supplementary Figure 1**).

### Lymphocyte Reconstitution in BM of MM Patients Post-ASCT

**Figure 1:**
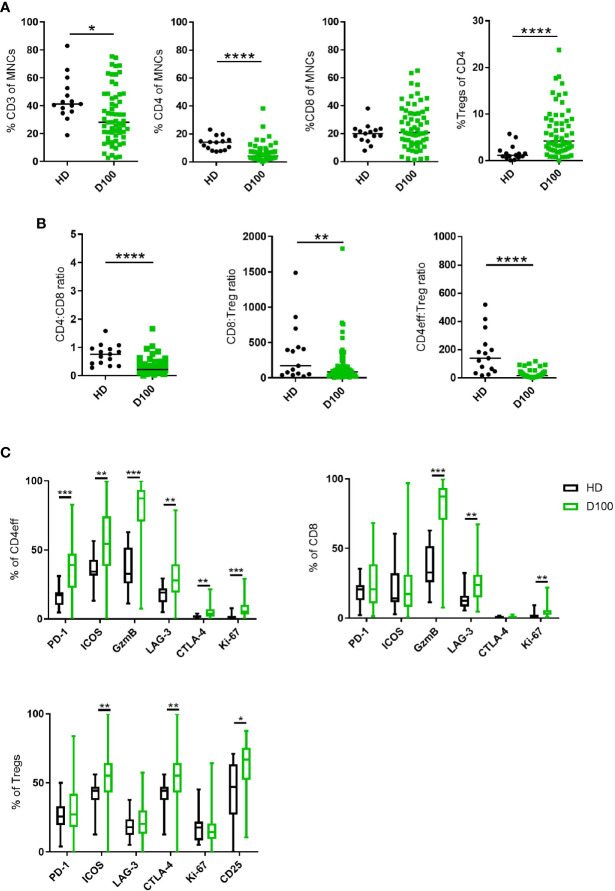
T cell subsets in MM BM post-ASCT. **(A)** Frequency of CD3, total CD4, CD8 T cells and Tregs. **(B)** CD4:CD8 and effector:Treg ratios calculated using absolute numbers of populations in each sample. **(C)** Frequency of markers of immune regulation, activation and proliferation on CD4eff, CD8, and Tregs. N=61. Statistical analysis by using Mann-Whitney U test *p < 0.05, **p < 0.01, ***p < 0.001. Lines indicate median for **(A, B)**. Box & whisker plots, **(C)**.

When T cell phenotypes in the post-ASCT BM of these patients were compared to those of healthy donors (HD), we observed that the frequency of total CD3 T cells in MM patients was lower (median 28% of live BM MNCs, vs 41.2% in HD; p=0.012). This was primarily due to a reduction in CD4 T cells (median 4.12% vs HD 14%, p=<0.0001) as there was no difference in the frequency of CD8 T cells (**Figure 1A**). Thus, the CD4:CD8 ratio was lower in MM patients when compared with HD (median 0.21 vs 0.76; p=<0.0001, **Figure 1B**).

We then differentiated CD4 T cells according to FoxP3 expression with CD4+FoxP3+ cells being T regulatory cells (Tregs) and CD4+FoxP3- cells as CD4 cells with effector potential, herein termed CD4eff for short. There was an increased frequency of BM Tregs (representative gating in **Supplementary Figure 2A**) in MM patients post-ASCT compared to HD (median 0.23% of live MNCs, vs HD 0.07%; p=0.02, when expressed as % of CD4 cells, median 4.2%, vs HD 1.13%, p<0.0001) (**Figure 1A**). This resulted in significantly lower CD4eff:Treg ratio (median 16.8 vs HD 140.2, p=<0.0001) and CD8:Treg ratio (median 46.9 vs HD 170.4, p=<0.01) when compared with HD. It should be noted here that the cohort of HD was younger with median age 36 years (27–60) compared to 58 years (36–71) in the patient cohort, thus these data remain to be confirmed in age-matched individuals.

In order to understand the immune landscape in the post-ASCT marrow, key markers of immune regulation, activation and proliferation on CD4eff and CD8 T cells were examined by flow cytometry (Representative FACS gating in **Supplementary Figure 2B**). These proteins were highly expressed on T cell subsets in the BM of post-ASCT MM patients compared to HD. CD4eff post-ASCT expressed higher frequencies of PD-1, LAG-3, ICOS, CTLA-4, GzmB, and Ki-67 (p=<0.0001, p=0.0016, p=0.0018, p=0.003, p=<0.0001, p=<0.0001 respectively) (**Figure 1C**). CD8 T cells in MM patients post-ASCT expressed higher frequencies of LAG-3, GzmB and Ki-67 (p=0.0003, p<0.0001, and p<0.0001 respectively) (**Figure 1C**). BM Tregs post-ASCT expressed higher frequencies of ICOS, CTLA-4, and CD25 compared with HD (p=0.0025, p=0.0025, and p=0.035 respectively) (**Figure 1C**).

### Markers of Immune Regulation, Activation, and Proliferation on Effector T Cells and Correlations With Timing of Relapse

We explored associations of T cell immune phenotype with clinical outcomes by comparing the PFS of patients expressing high or low frequency (> or ≤ median respectively) of T cell subsets, or markers of immune regulation, activation and proliferation (PD-1, LAG-3, ICOS, CTLA-4, GzmB, and Ki-67) on T cell subsets. We found no association of frequencies of CD4eff, CD8, or Tregs with PFS (**Supplementary Figure 3**).

**Figure 2:**
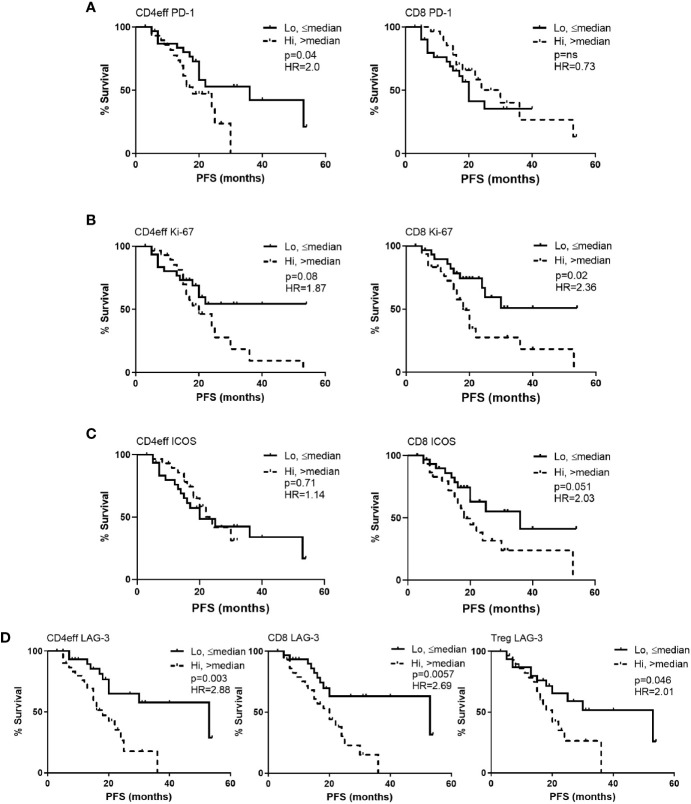
Kaplan-Meier estimates of progression free survival (PFS) according to frequency of PD-1, Ki-67, ICOS and LAG-3 positivity in CD4eff, CD8 and Treg cells in bone marrow of MM patients at D100 post ASCT. High refers to frequency that is above median, and low equal to or below median of **(A)** PD-1, **(B)** Ki-67, **(C)** ICOS, and **(D)** LAG-3 on T cell subsets as indicated. CD4eff PD-1 refers to the % of CD4eff that are PD-1 positive, Treg LAG-3 refers to the % of Treg that are LAG-3 positive, etc. Statistical analysis by Log-Rank.

When examining individual markers of immune regulation, activation, and proliferation we observed that MM patients with high frequency of PD-1 (>median) on CD4eff (CD4 PD-1^hi^) had significantly shorter PFS (median 18 months vs 36 for those with low frequency of PD-1 (≤ median) on CD4eff, p=0.042) (**Figure 2A**). In contrast, frequency of PD-1 on CD8 T cells was not associated with PFS (p=0.73), but Ki-67 frequency was. MM patients with high frequency of Ki-67 (>median) on CD8 cells had significantly shorter PFS (median 18 months vs 27 in the case of low frequency of Ki-67; p=0.048) (**Figure 2B**). A trend toward shorter PFS was seen in association with CD4eff with high frequency of Ki-67 (p=0.08, **Figure 2B**). There was also an association of CD8 ICOS^hi^ with poorer outcomes (median 18 months PFS vs 36 months for CD8 ICOS^lo^, p=0.051) (**Figure 2C**).

LAG-3 expression on all three T cell subsets correlated with shorter PFS. Comparing CD4 LAG-3^hi^ vs CD4 LAG-3^lo^, median PFS was 18 months vs 53 respectively (p=0.003), CD8 LAG-3^hi^ vs CD8 LAG-3^lo^ median was 20 months vs 53 respectively (p=0.006) and Treg LAG-3^hi^ vs Treg LAG-3^hi^, median was 18 months vs 53, (p=0.046, **Figure 2D**). Frequencies of LAG-3 were positively correlated amongst the different T cell subsets; (CD4eff LAG-3+ vs CD8 LAG-3+ r=0.82, p<0.0001; CD4eff LAG-3+ vs Treg LAG-3+, r=0.56, p<0.0001; Treg LAG-3+ vs CD8 LAG-3+, r=0.5, P<0.0001, **Supplementary Figure 4**).

A multivariate Cox regression model was built including ISS, depth of response, and the immune phenotypes (CD4eff PD-1+, CD8 Ki-67+, and Treg LAG-3+) which were found to correlate with PFS on univariate analysis. CD4eff LAG-3+ and CD8 LAG-3+ were omitted from the multivariable analysis to avoid problems relating to multicollinearity. In this model, high frequency of PD-1 on CD4eff retained independent prognostic value, along with Ki-67 on CD8 T cells and ISS stage (**Table 1**).

**Table 1:** Univariate and multivariate analysis.

	Univariate			Multivariate		
	HR	CI	P values	HR	CI	P values
**Response**	2.77	1.64–10.07	0.003	1.23	0.88–1.73	0.232
**ISS stage**	3.67	1.29–8.22	0.013	0.24	0.077-0.75	0.014
**Tregs LAG-3+**	2.01	1.04–4.44	0.046	1.35	0.55–3.29	0.508
**CD4eff PD-1+**	1.99	1.07–4.75	0.042	3.55	1.39–9.07	0.008
**CD8 Ki-67+**	2.36	1.17–4.93	0.047	1.35	0.55–3.29	0.007

**Figure 3:**
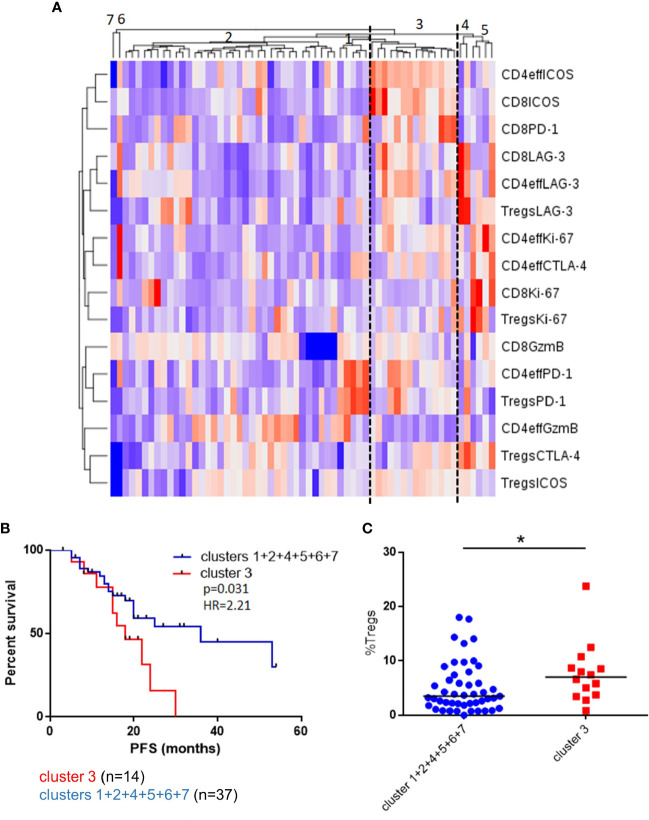
Unsupervised clustering of BM T cell phenotype defines a patient subset with a distinct phenotype and poorer outcomes. **(A)** Unsupervised hierarchical clustering and heatmap of activation and inhibitory receptors on CD4eff, CD8, and Treg cells in 61 patients 3 months post-ASCT. **(B)** PFS in patients in cluster 3 and clusters 1 + 2+4+5+6+7+8. **(C)** Frequency of Tregs in patients in cluster 3 and other clusters. Statistical analysis by using Mann-Whitney U test *p < 0.05.

To explore these concepts further and appreciating that high frequencies of CD4eff PD-1+ and CD8 Ki-67+ are likely markers for a broader immune phenotype characterizing suboptimal anti-tumor immunity, we undertook an unsupervised analysis of single proteins on each T cell subset. We identified a patient group consisting of 14/61 (23%) patients characterized by high frequencies of PD-1 and ICOS on Tregs, CD4eff and CD8 cells and high LAG-3 expression on CD4eff and CD8 (Cluster 3, **Figure 3**). Detailed analysis of marker expression on T cell populations in Cluster 3 compared to rest of cohort is illustrated in **Supplementary Figure 5**. Eleven of the 14 patients in this group were also classed as CD4eff PD-1^hi^ on previous single marker analysis and interestingly while high frequency of Ki-67 on CD4eff was associated with this high risk group, CD8 Ki-67+ was not.

While these findings are preliminary and remain to be confirmed in larger cohorts, they suggest that the immune function in the early post-ASCT marrow may be an important indicator of longer term outcomes, either by directly exerting anti-tumor control, or as a surrogate marker for host fitness.

### BM T Cell Subsets and Phenotype in Post-ASCT Marrow Compared to Baseline

We asked whether the T cell phenotypes that we observed in the post-transplant marrow were reflective of diagnostic immune profiles. Using a more detailed FACs panel, we compared the post-ASCT BM to that of baseline pre-induction BM using paired samples from a separate cohort of nine patients (**Supplementary Table 2**). Compared to the healthy donors (from **Supplementary Table 1**) and in keeping with our previous report (15), there was a higher frequency of Tregs as a proportion of CD4 cells and increased CD8:Treg and CD4eff:Treg ratios in baseline BM samples (**Supplementary Figure 6**).

**Figure 4:**
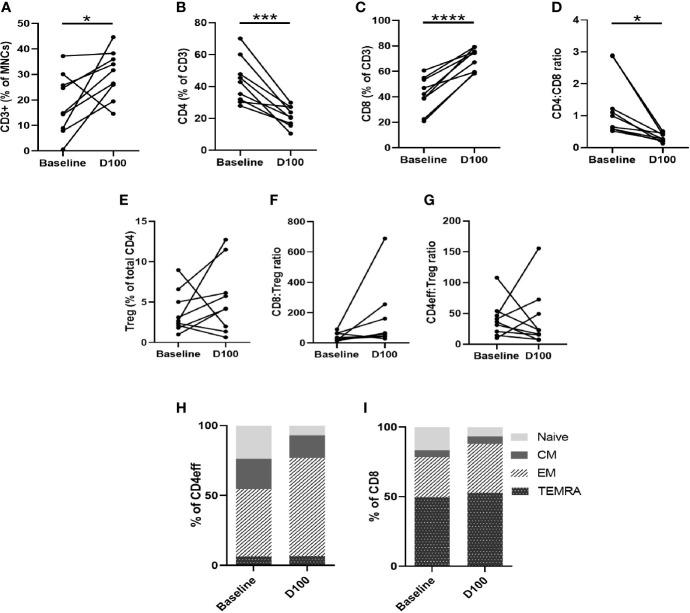
Characterization of T cell subsets by flow cytometry of paired BM samples at diagnosis and D100 post-ASCT. **(A)** Change in frequency of total CD3, **(B)** CD4, **(C)** CD8, **(D)** CD4:CD8 ratio, **(E)** Tregs, **(F)** CD8:Treg ratio **(G)** CD4:Treg ratio. Ratios calculated using absolute numbers of populations in each sample. **(H)** Changes in CD4eff memory subtypes as defined by CCR7 and CD45RA expression, **(I)** CD8 memory subtypes. Statistical analysis by paired Student’s t-tests *p < 0.05, ***p < 0.001, ****p < 0.0001.

Comparing paired baseline and D100 BM, we observed that, as a proportion of BM MNCs, CD3 cell numbers were higher at D100 post-ASCT (p=0.039 compared to baseline, **Figure 4A**). The frequency of CD4 cells decreased (p=0.0006) (**Figure 4B**) while that of CD8 cells increased (p<0.0001) (**Figure 4C**), resulting in a reduction in CD4/CD8 ratio (p=0.012) (**Figure 4D**). There were no significant differences in Tregs (**Figure 4E**), CD8:Treg ratio (**Figure 4F**) or CD4eff:Treg ratio (**Figure 4G**) between diagnostic and post-ASCT.

We next examined changes in CD3, CD4, and CD8 memory phenotype between baseline and D100 post-ASCT. Using CCR7 and CD45RA to differentiate memory phenotypes (**Supplementary Figure 7A**), we observed a decrease in naïve T cells (p= 0.0011 by paired Student t test) and increase in effector memory (EM) (p=0.0026) CD4eff (**Figure 4H**). In the CD8 compartment, there was also a reduction in naïve cells (p=0.028), and increases in CD8 EM and TEMRA (effector memory T cells re-expressing CD45RA) that did not reach statistical significance (**Figure 4I**). Overall, therefore, post-ASCT, there was a shift away from naïve and towards a more differentiated effector phenotype in both CD4eff and CD8 cells which is broadly consistent with previous studies on the peripheral blood of post-ASCT MM patients (9, 16). When comparing with diagnostic samples, though, some contribution of the induction chemotherapy cannot be ruled out as we did not examine the marrow pre-ASCT.

Next, we examined the expression of immune regulation, activation and proliferation proteins on the different T cell subsets, including PD-1, LAG-3, ICOS, GzmB, CTLA-4, Ki-67. We observed that PD-1 on CD4eff was significantly higher at D100 post-ASCT, compared to baseline (p=0.0004) (**Supplementary Figure 7D**). This finding is consistent with a previous report on peripheral blood (9). We also found the frequencies of Ki-67 and GzmB on CD4eff to be increased in the post-ASCT BM (p=0.036, p=0.0016 respectively, **Supplementary Figures 7E, F**). These findings are suggestive of increased immune activation in the post-transplant marrow, although we found no differences in LAG-3, ICOS and CTLA-4. In the CD8 subset, the only significant change we found was an increase in GzmB post-ASCT (p=0.0016) (**Supplementary Figures 7G, H**), again suggesting increased immune activation. Dimensionality reduction analysis was undertaken of the CD3 compartment for three paired samples analysed in a single run, this confirmed a broadly more differentiated phenotype post-ASCT, with a reduction in CCR7 and CD27 and an increase in Granzyme B expression (**Supplementary Figure 8**).

**Figure 5:**
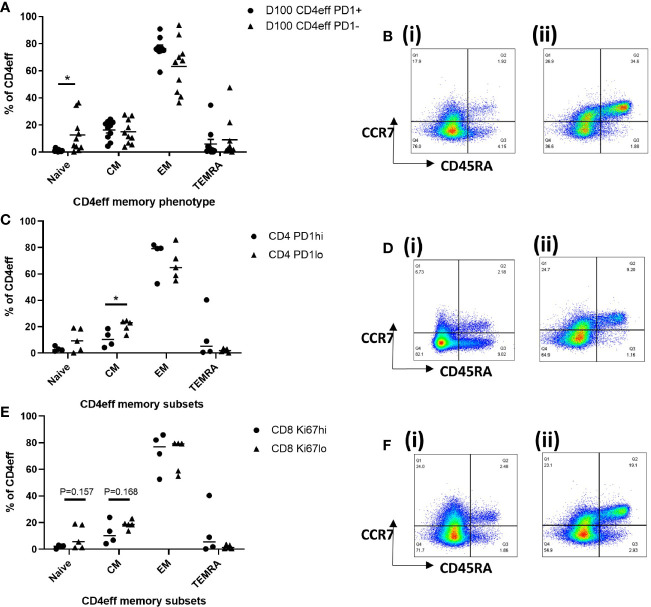
Characterization of T cell memory subsets by flow cytometry at D100. **(A)** Graph of memory phenotypes of CD4eff PD-1+ vs CD4eff PD-1- populations. **(B)** Flow plots of a single sample showing memory phenotype of (i) CD4eff PD-1+ and (ii) CD4eff PD-1-. **(C)** CD4 memory phenotype of patients with high (frequency that is above median, n=4) or low (frequency that is equal to and below median, n=5) frequency of CD4eff PD-1+. **(D)** Flow plots showing memory phenotype of CD4eff from representative patients with (i) high and (ii) low frequency of CD4eff PD-1+ cells. **(E)** CD4 memory phenotype of patients with high or low frequency of CD8 Ki-67+ cells; **(F)** Flow plots showing CD4eff memory phenotype from representative patients with (i) high and (ii) low frequency of CD8 Ki-67+. Median indicated. Statistical analysis by Student *t*-test *p < 0.05, paired analysis in **(A)**.

We then considered whether the presence of high numbers of CD4eff PD-1+, and CD8 Ki-67+ cells at D100 might be indicative of a broader immune differentiation phenotype. Thus, we examined the differentiation status of CD4eff, comparing PD-1+ with PD-1-populations. We found that the CD4eff PD-1+ populations contained less naïve CD4eff cells (p=0.019) and more CD4eff EM (p=0.049) (**Figures 5A, B**) compared to CD4eff PD-1- cells. We also observed that CD8 Ki-67+ populations contained more EM (p=0.007) as well as less TEMRA cells (0.0001) (**Supplementary Figure 9A**). We then asked whether patient samples with a higher percentage of CD4eff expressing PD-1 had a more differentiated T cell phenotype. Defining CD4eff PD-1^hi^ patients as >median %PD-1+ among CD4eff, we observed that these patients had relatively lower proportion of CD4eff central memory (CM) (p=0.03) (**Figures 5C, D**), when compared with BM from patients with CD4eff PD-1^lo^(≤median %PD-1+ CD4eff). When we examined the CD4eff differentiation phenotype in patients with higher frequencies of CD8 Ki-67+ (>median Ki-67+), we observed a trend towards lower proportions of naïve and CM CD4eff compared to patients with lower frequencies of CD8 Ki-67+ **Figures 5E, F**). Interestingly, there was no significant difference in CD8 memory phenotype in any of these comparisons (**Supplementary Figures 10A, B**).

Taken as a whole, the T cell compartment in post-ASCT BM is more differentiated with features of immune activation. Whether this is directly related to the transplant or is a post-treatment effect remains to be established. Our data also suggest that distinct features of late differentiation and activation are correlated with clinical outcomes.

### T Cell Receptor Repertoire Post-ASCT Compared to Baseline

Hypothesizing that immunological differences that are correlated to timing of relapse may reflect anti-tumor immune responses, we asked if such responses might also be reflected in particular changes in the T cell receptor (TCR) repertoire post-ASCT. We undertook TCR sequencing of paired BM MNCs at baseline and post-ASCT from five patients (clinical characteristics in **Supplementary Table 4**). We first assessed the diversity of the repertoires at BL and post-ASCT. In general, there were greater numbers of reads in baseline samples (**Supplementary Figure 11**) which were thus iteratively subsampled as outlined in methods. In one case (α-chain of T-04), number of post-ASCT reads exceeded BL reads, thus the former was subsampled.

**Figure 6:**
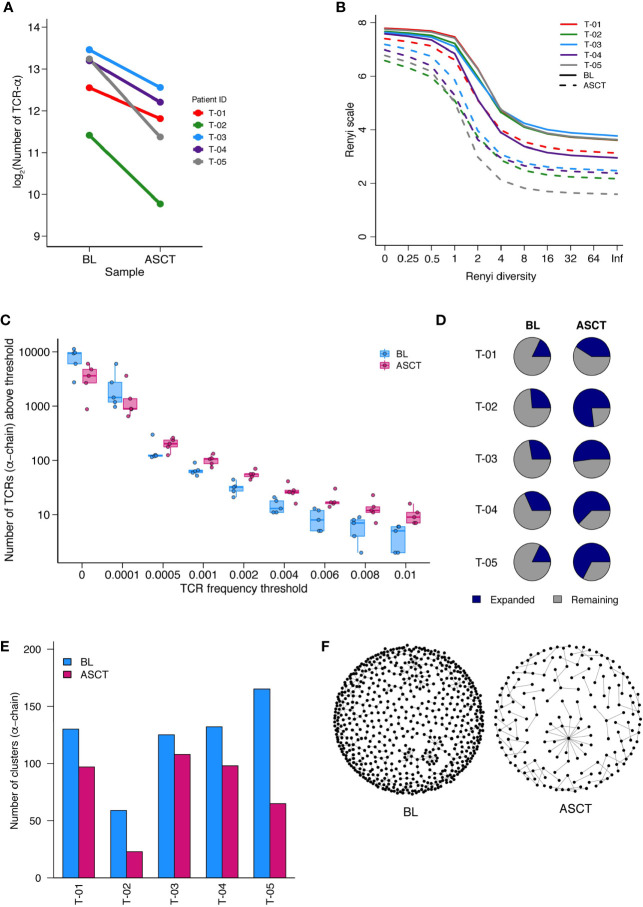
TCR-Sequencing analysis of five paired patient samples at baseline (BL) and post-ASCT. **(A) **Number of α-chain TCR sequences collected at BL and D100 post-ASCT. Adjusted p=0.0052 by paired t-test. **(B)** Diversity of repertoires calculated as Renyi entropy values from 0 to infinity for the α-chain for each patient sample. Each repertoire was sub-sampled to 3000 TCRs 100 times. **(C)** Number of α-chain sequences detected above a given frequency threshold. Minimum and maximum values are indicated by extreme points and the thick horizontal line in the middle indicates the median value. **(D)** Pie-chart for each patient sample depicting the proportion of expanded (frequency >1/1,000) and unexpanded α-chain TCRs. **(E)** Clustering analysis then performed as a measure of sequence similarity between unique TCRs analysis on the basis of triplet amino acid sharing between two CDR3 sequences. Number of clusters from top 50 expanded α-chain CDR3 sequences per sample. **(F)** Representative image (T-05) of α-chain CDR3 clustering analysis. (Similarity threshold 0.80).

The number of unique TCR sequences (repertoire richness) was consistently reduced post-ASCT (**Figure 6A**, **Supplementary Figure 12A**). The Renyi diversity series captures a range of diversity metrics, which include the Shannon diversity (Renyi index = 1) and the Simpson’s diversity index (Renyi index = 2). The diversity of the samples was consistently reduced post-transplant, across the whole range of Renyi indices, and in all patients (**Figure 6B**, **Supplementary Figure 12B**). This reduction in diversity could be attributed to an effect of treatment, including the transplant in all samples. In addition to this, we also observed an increased number of expanded TCR sequences post-transplant (above a frequency threshold of 1/1,000) (**Figure 6C**, **Supplementary Figure 12C**). We further detected an increase in the proportion of expanded TCRs post-ASCT (**Figure 6D**, **Supplementary Figure 12D**). These findings suggest the presence of a narrower and more focused persistent TCR repertoire in the BM of MM patients following transplantation.

We next looked for the presence of related TCRs by the presence of similar, overlapping CDR3 amino acid sequences (14). In support of a more narrow and targeted T cell response post-ASCT, there were fewer clusters of CDR3 sequences in all samples (**Figure 6E**, **Supplementary Figure 12E**, **Supplementary Table 5**. A representative paired sample is illustrated in **Figure 6F**). We next focused on shared TCR sequences in paired BL and D100 samples (**Supplementary Figure 12F**). We asked what proportion of expanded TCRs in the post-ASCT samples were present at baseline. For two patients, well over half of expanded TCR sequences post-ASCT were present at baseline suggesting a remarkable stability in the intertumoral niche (T-03 and T-04). Two other patients seem to have a more dynamic repertoire (T-01 and T-05) and analysis of one patient was limited due to the few TCRs (T-02).

## Discussion

In this work carried out exclusively on BM T cell populations, we present data describing the increased activation status of both regulatory and effector T cells following ASCT for MM, and identified an association between high expression of immune markers and increased risk of relapse. Our data indicate that the post-ASCT marrow is characterized by a reduction in CD4, but an increase in CD8 cells, leading to lower CD4:8 ratios. Treg cells are generally increased in MM patients compared with HD, whether at diagnosis or post-ASCT. When compared with diagnostic marrow, there is a general increase in EM and a reduction in naïve subsets of both CD4eff and CD8 cells. All 3 T cell subsets show evidence of activation in the post-ASCT marrow, with high level expression of several markers of immune regulation, activation and proliferation, including PD-1, ICOS, CTLA-4, GzmB, LAG-3, Ki-67 on CD4 eff cells, LAG-3, GzmB, and Ki-67 on CD8 cells and ICOS, CTLA-4, and CD25 on Treg cells. With respect to clinical outcomes, we used unsupervised analysis to identify a patient subgroup at greater risk of early relapse (p=0.031) whose BM contained higher frequencies of Tregs, and greater co-activation and co-inhibitory marker (ICOS, PD-1, LAG-3) expression on Tregs, CD4eff, and CD8 cells. Using single feature analysis, we also observed that higher frequencies of CD4eff PD-1+ and CD8 Ki-67+ cells in the post-ASCT marrow were independently associated with inferior progression free survival. Finally, studying paired diagnostic and post-ASCT BM from 5 patients, we note reduced abundance of TCR sequences with a greater proportion of high frequency or expanded sequences indicating a more focused and persistent TCR repertoire in the post-transplant BM of MM. Intriguingly in two patients, we observed that over half of expanded TCRs found in post-ASCT BM were present at diagnosis, prior to start of chemotherapy. We did not conduct similar analyses on pre-transplant BM samples and hence the differences we observe, in comparison with diagnostic, pre-treatment samples, are the combined result of induction therapy, a reduction in tumour burden, and high dose alkylator therapy with stem cell re-infusion. Where, in subsequent passages, we refer to the effect of ASCT, this should be borne in mind.

To our knowledge, this is the first report of the immune profile of T cells from the post-transplant BM in a large cohort of MM patients. Analysis of BM T cells as opposed to circulating cells from peripheral blood is likely to yield greater insight into the tumor niche, given observed differences between circulating and BM resident T cells in disease (17, 18), and may also indicate earlier changes within the tumor immune microenvironment.

We identified two T cell features associated with poor outcome. We have recently described an association between high frequency of BM PD-1 expressing CD4eff cells at diagnosis and risk of relapse (15), corroborated by our findings here. Higher levels of PD-1 on CD4 T cells, usually taken to be an indicator of exhaustion, has been described in chronic infection, together with decreased expression of co-stimulatory molecules and reduced cytokine expression (19). An interesting observation we made here in the post-ASCT BM was an association between high frequency of Ki-67 on CD8 cells, and early relapse. Although greater frequencies of CD8 Ki-67+ cells have been associated with superior outcomes in the context of checkpoint blockade in melanoma (20), emerging data suggest that early dysfunctional cells are the major intratumorally proliferating (and Ki-67 expressing) compartment (21) suggesting that high frequency of CD8 Ki-67+ may be indicative of T cell dysfunction. Interestingly, we found there was also a trend for an association between CD4 Ki-67+ frequency with clinical outcome.

In this work, we also identified a group of patients, making up just under a quarter of the cohort, sharing an immune profile and a shorter PFS. That immune profile was characterized by high frequencies of the co-activation/co-inhibitory proteins PD-1, ICOS, and LAG-3 on Treg as well as CD4eff and CD8 cells, although did not include patients who expressed the very highest frequencies of CD4 PD-1+ (found in cluster 4), CD8 Ki-67+ (cluster 5), or LAG-3(cluster 4), suggesting the limitations of single feature analysis. Further in-depth study of a larger patient cohort is needed to comprehensively characterize immune profiles associated with clinical outcomes. This would provide the basis for rational design of immunotherapies to optimize immune fitness and function in the BM of MM patients following ASCT (22).

Further analysis of a small number of patient BM samples with a more detailed FACS panel, and by performing intra- and inter- patient comparisons of T cell phenotypes, we observed an association between high frequency of CD4 PD-1+ or CD8 Ki-67+ and more differentiated memory phenotypes (variably less naïve or central memory and more EM). This further supports the notion that these immune markers are part of a broader immune profile that also includes T cell memory phenotype. Indeed a high proportion of central memory cells in adoptively transferred T cells have been strongly correlated with persistence and patient outcomes (23). CD4 memory phenotypes, in particular, a high CM: EM, have been found to correlate with poorer prognosis in other tumor types (24–28).

An expansion of effector and memory subsets post-ASCT is consistent with a reduction in clonal diversity post-transplant, proportionally more expanded clones and with less TCR sequence clustering. This reduction in TCR diversity has been described previously on analysis of PB T Cells post-ASCT carried out for multiple sclerosis (29). To our knowledge this is the first report of paired TCR sequencing of BM T cells in MM patients undergoing ASCT. In two of the five patients, there was notable stability of TCR sequences in the tumoral niche with the majority of (post-ASCT) expanded TCR sequences detected in the baseline sample. If the expanded BM T cell clones represent an anti-tumor population of T cells, the preservation of this population despite extensive destruction of the repertoire during the transplant process may be an important indicator of the ability of the immune response to control the growth of surviving tumor cells post-transplant. However, a greater number of samples are needed to discern if repertoire overlap, diversity, or clustering are associated with outcome. Application of single cell sequencing to allow TCR alpha/beta pairing may facilitate an investigation of the tumor specificity of expanded TCR in the BM post-ASCT.

In summary, we observe an increase in activated and memory T cells characterized by upregulation of receptors associated with antigen engagement including co-stimulatory receptors and co-inhibitory receptors that associate with relapse risk post-ASCT. Pending confirmation in larger series, in the post-ASCT settings, the minimal disease burden and re-constitution of the immune system may be a favorable context for immunotherapies, including cellular therapies and checkpoint blockade. Detailed insight into the immune microenvironment post-ASCT is thus needed and is a prerequisite to designing rational therapeutic strategies to optimize the tumor-targeting immune response in MM patients post transplantation.

## Data Availability Statement

The original contributions presented in the study are included in the article/**Supplementary Material**. Further inquiries can be directed to the corresponding authors.

## Ethics Statement

The studies involving human participants were reviewed and approved by REC reference: 07/Q0502/17, Health Research Authority, UK. The patients/participants provided their written informed consent to participate in this study.

## Author Contributions

KY, SQ, and BC designed this work. NA, LL, GK, HR, LC, WW, MD, JHen and JHer acquired and/or analyzed the data. SC, KY, and LL provided clinical correlates. EF, MC, DG-F, SC and HR collected and or processed the clinical samples for analysis. LL, KY, and NA wrote this manuscript. All authors contributed to the article and approved the submitted version.

## Funding

NA's PhD studentship was funded by King Faisal Specialist and Research Centre. LL is a Clinician Scientist funded by the Medical Research Council (Clinician Scientist Fellow, MR/S001883/1). GK is funded by Cancer Research UK. LC was funded by the Cancer Research UK-University College London (CRUK-UCL) Centre Award [C416/A25145]. SQ is a Cancer Research UK Senior Cancer Research Fellow. KY receives funding from UCLH Biomedical Research Centre.

## Conflict of Interest

The authors declare that the research was conducted in the absence of any commercial or financial relationships that could be construed as a potential conflict of interest.
