# Supplementary material for: Increased Immune-Regulatory Receptor Expression on Effector T Cells as Early Indicators of Relapse Following Autologous Stem Cell Transplantation for Multiple Myeloma
Source: Front Immunol. 2021 Feb 25;12:618610. doi: 10.3389/fimmu.2021.618610 (PMC7946836; doi:10.3389/fimmu.2021.618610)
Supplement: Supplementary file 1 [file Presentation_1.pptx]

## Slide 1
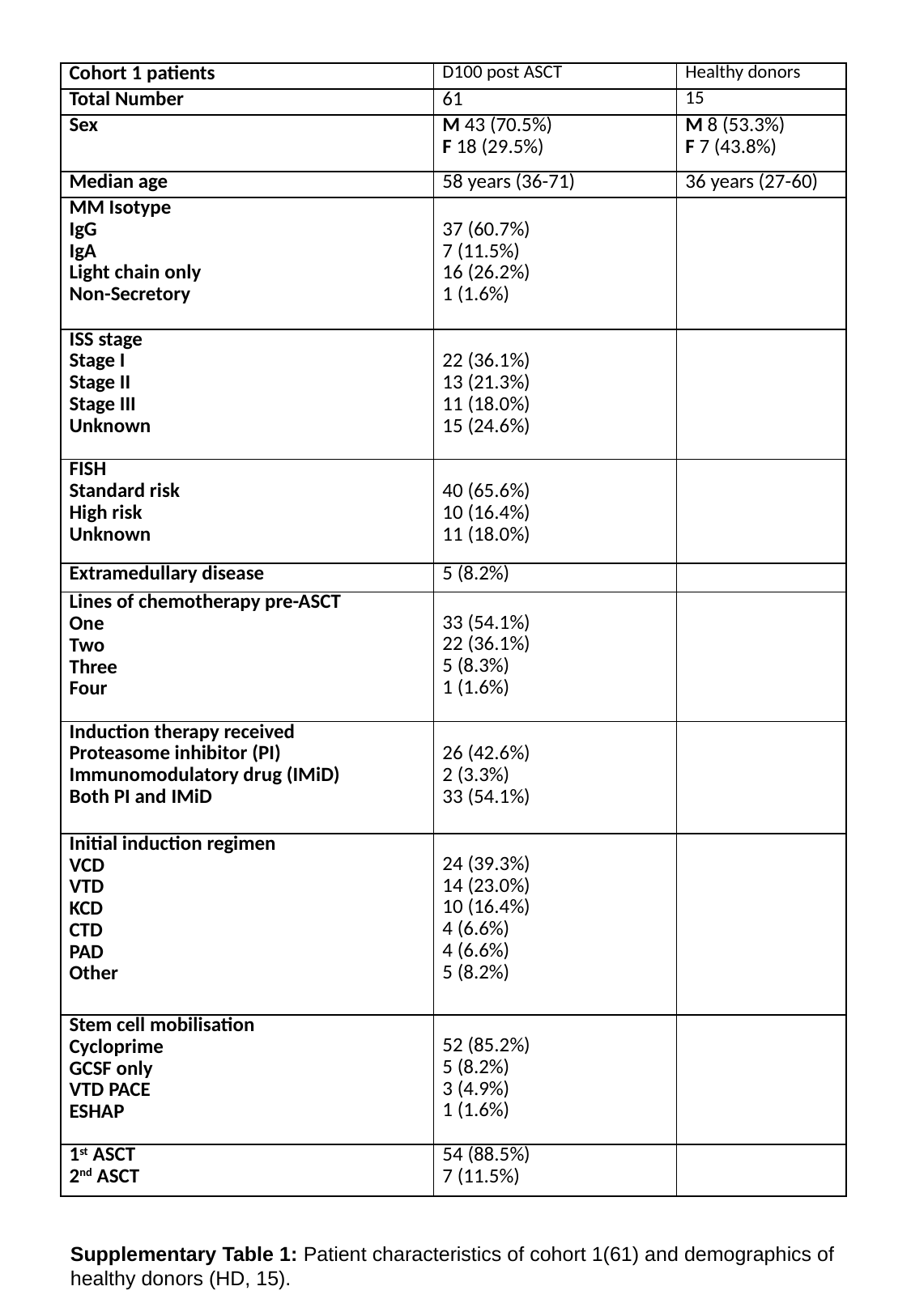

| Cohort 1 patients | D100 post ASCT | Healthy donors |
| --- | --- | --- |
| Total Number | 61 | 15 |
| Sex | M 43 (70.5%) F 18 (29.5%) | M 8 (53.3%) F 7 (43.8%) |
| Median age | 58 years (36-71) | 36 years (27-60) |
| MM Isotype IgG IgA Light chain only Non-Secretory | 37 (60.7%) 7 (11.5%) 16 (26.2%) 1 (1.6%) | |
| ISS stage Stage I Stage II Stage III Unknown | 22 (36.1%) 13 (21.3%) 11 (18.0%) 15 (24.6%) | |
| FISH Standard risk High risk Unknown | 40 (65.6%) 10 (16.4%) 11 (18.0%) | |
| Extramedullary disease | 5 (8.2%) | |
| Lines of chemotherapy pre-ASCT One Two Three Four | 33 (54.1%) 22 (36.1%) 5 (8.3%) 1 (1.6%) | |
| Induction therapy received Proteasome inhibitor (PI) Immunomodulatory drug (IMiD) Both PI and IMiD | 26 (42.6%) 2 (3.3%) 33 (54.1%) | |
| Initial induction regimen VCD VTD KCD CTD PAD Other | 24 (39.3%) 14 (23.0%) 10 (16.4%) 4 (6.6%) 4 (6.6%) 5 (8.2%) | |
| Stem cell mobilisation Cycloprime GCSF only VTD PACE ESHAP | 52 (85.2%) 5 (8.2%) 3 (4.9%) 1 (1.6%) | |
| 1st ASCT 2nd ASCT | 54 (88.5%) 7 (11.5%) | |
Supplementary Table 1: Patient characteristics of cohort 1(61) and demographics of healthy donors (HD, 15).

## Slide 2
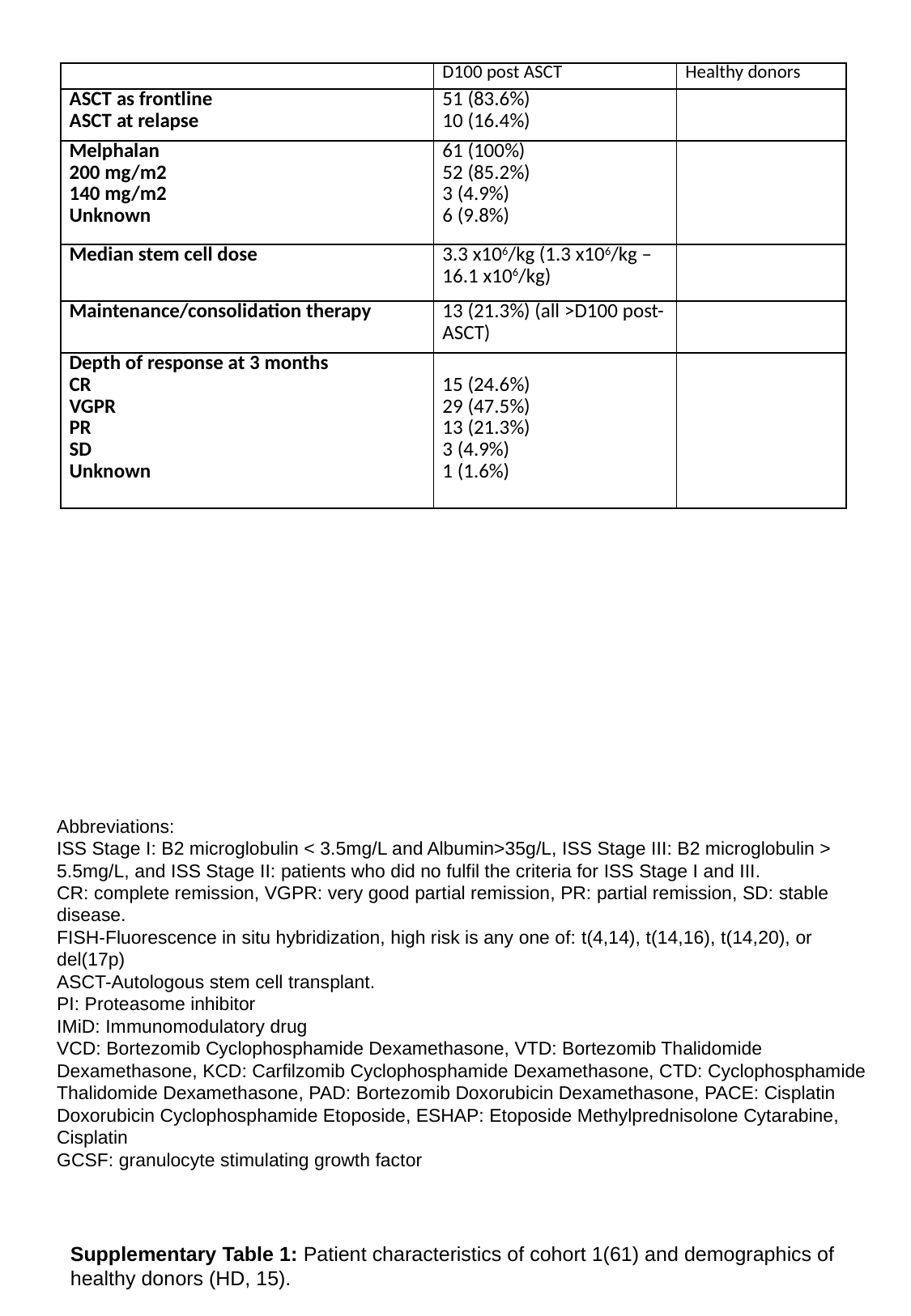

| | D100 post ASCT | Healthy donors |
| --- | --- | --- |
| ASCT as frontline ASCT at relapse | 51 (83.6%) 10 (16.4%) | |
| Melphalan 200 mg/m2 140 mg/m2 Unknown | 61 (100%) 52 (85.2%) 3 (4.9%) 6 (9.8%) | |
| Median stem cell dose | 3.3 x10^6^/kg (1.3 x10^6^/kg – 16.1 x106/kg) | |
| Maintenance/consolidation therapy | 13 (21.3%) (all >D100 post-ASCT) | |
| Depth of response at 3 months CR VGPR PR SD Unknown | 15 (24.6%) 29 (47.5%) 13 (21.3%) 3 (4.9%) 1 (1.6%) | |
Abbreviations:
ISS Stage I: B2 microglobulin < 3.5mg/L and Albumin>35g/L, ISS Stage III: B2 microglobulin > 5.5mg/L, and ISS Stage II: patients who did no fulfil the criteria for ISS Stage I and III.
CR: complete remission, VGPR: very good partial remission, PR: partial remission, SD: stable disease.
FISH-Fluorescence in situ hybridization, high risk is any one of: t(4,14), t(14,16), t(14,20), or del(17p)
ASCT-Autologous stem cell transplant.
PI: Proteasome inhibitor
IMiD: Immunomodulatory drug
VCD: Bortezomib Cyclophosphamide Dexamethasone, VTD: Bortezomib Thalidomide Dexamethasone, KCD: Carfilzomib Cyclophosphamide Dexamethasone, CTD: Cyclophosphamide Thalidomide Dexamethasone, PAD: Bortezomib Doxorubicin Dexamethasone, PACE: Cisplatin Doxorubicin Cyclophosphamide Etoposide, ESHAP: Etoposide Methylprednisolone Cytarabine, Cisplatin
GCSF: granulocyte stimulating growth factor
Supplementary Table 1: Patient characteristics of cohort 1(61) and demographics of healthy donors (HD, 15).

## Slide 3
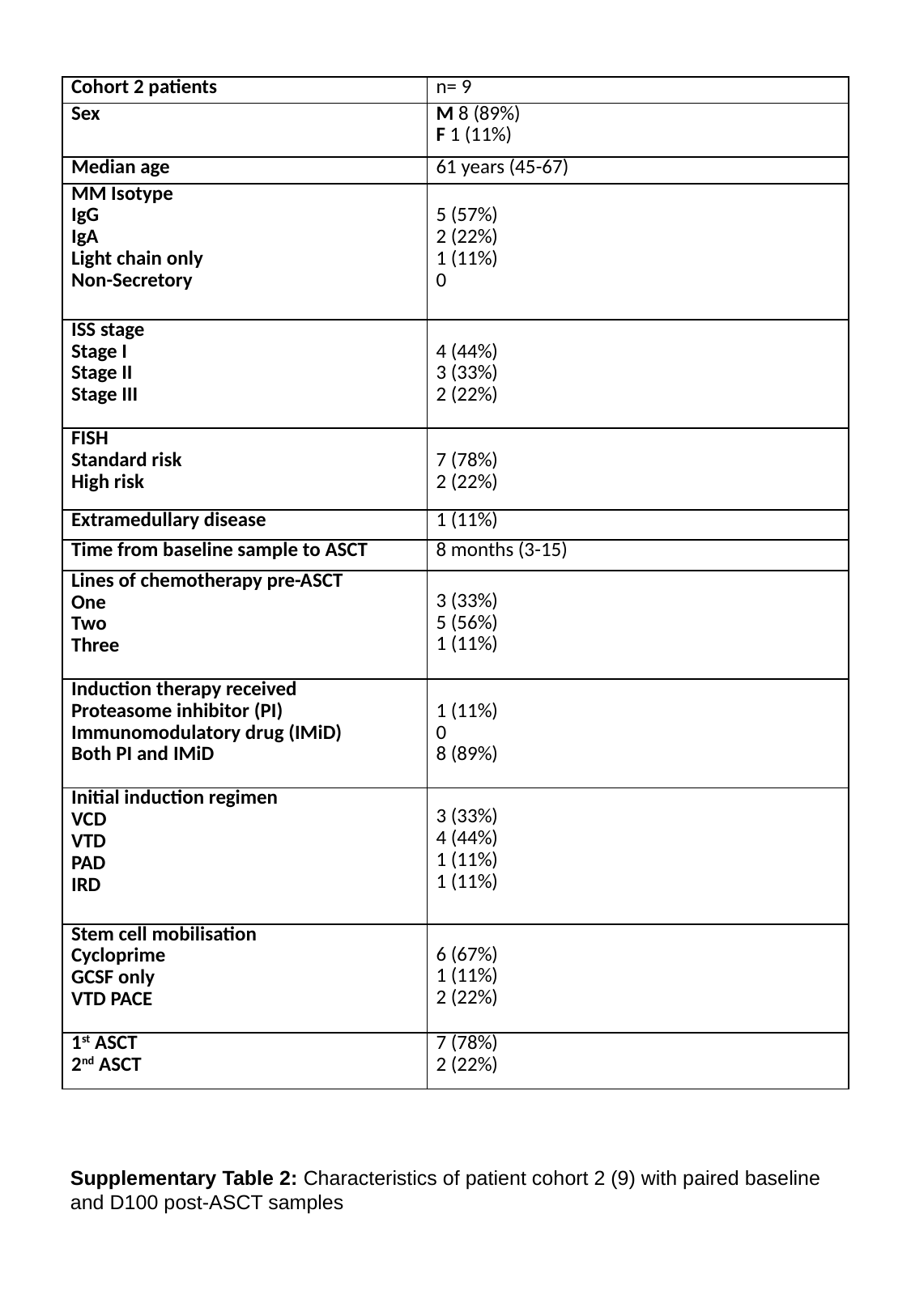

| Cohort 2 patients | n= 9 |
| --- | --- |
| Sex | M 8 (89%) F 1 (11%) |
| Median age | 61 years (45-67) |
| MM Isotype IgG IgA Light chain only Non-Secretory | 5 (57%) 2 (22%) 1 (11%) 0 |
| ISS stage Stage I Stage II Stage III | 4 (44%) 3 (33%) 2 (22%) |
| FISH Standard risk High risk | 7 (78%) 2 (22%) |
| Extramedullary disease | 1 (11%) |
| Time from baseline sample to ASCT | 8 months (3-15) |
| Lines of chemotherapy pre-ASCT One Two Three | 3 (33%) 5 (56%) 1 (11%) |
| Induction therapy received Proteasome inhibitor (PI) Immunomodulatory drug (IMiD) Both PI and IMiD | 1 (11%) 0 8 (89%) |
| Initial induction regimen VCD VTD PAD IRD | 3 (33%) 4 (44%) 1 (11%) 1 (11%) |
| Stem cell mobilisation Cycloprime GCSF only VTD PACE | 6 (67%) 1 (11%) 2 (22%) |
| 1st ASCT 2nd ASCT | 7 (78%) 2 (22%) |
Supplementary Table 2: Characteristics of patient cohort 2 (9) with paired baseline and D100 post-ASCT samples

## Slide 4
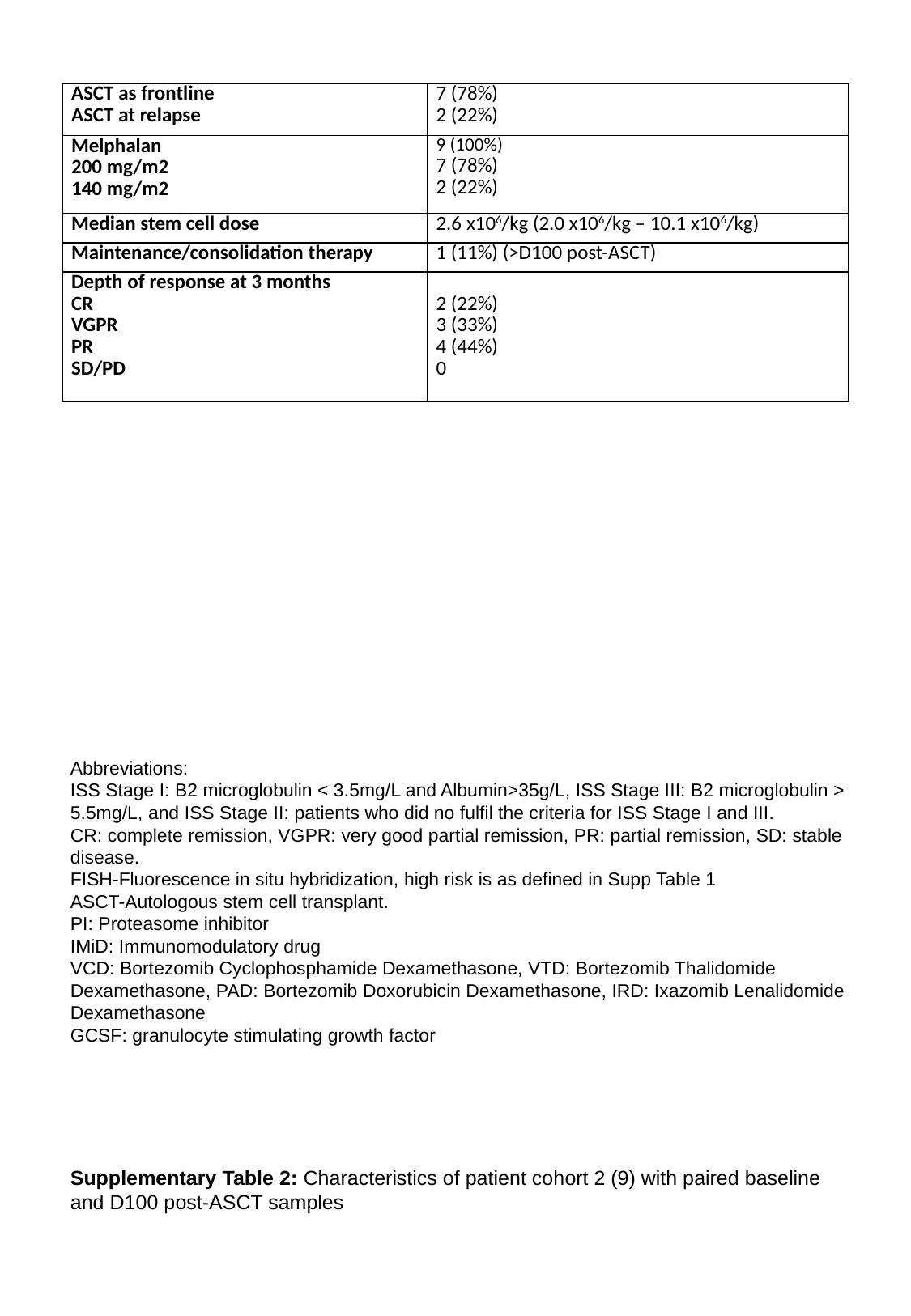

| ASCT as frontline ASCT at relapse | 7 (78%) 2 (22%) |
| --- | --- |
| Melphalan 200 mg/m2 140 mg/m2 | 9 (100%) 7 (78%) 2 (22%) |
| Median stem cell dose | 2.6 x10^6^/kg (2.0 x10^6^/kg – 10.1 x106/kg) |
| Maintenance/consolidation therapy | 1 (11%) (>D100 post-ASCT) |
| Depth of response at 3 months CR VGPR PR SD/PD | 2 (22%) 3 (33%) 4 (44%) 0 |
Abbreviations:
ISS Stage I: B2 microglobulin < 3.5mg/L and Albumin>35g/L, ISS Stage III: B2 microglobulin > 5.5mg/L, and ISS Stage II: patients who did no fulfil the criteria for ISS Stage I and III.
CR: complete remission, VGPR: very good partial remission, PR: partial remission, SD: stable disease.
FISH-Fluorescence in situ hybridization, high risk is as defined in Supp Table 1
ASCT-Autologous stem cell transplant.
PI: Proteasome inhibitor
IMiD: Immunomodulatory drug
VCD: Bortezomib Cyclophosphamide Dexamethasone, VTD: Bortezomib Thalidomide Dexamethasone, PAD: Bortezomib Doxorubicin Dexamethasone, IRD: Ixazomib Lenalidomide Dexamethasone
GCSF: granulocyte stimulating growth factor
Supplementary Table 2: Characteristics of patient cohort 2 (9) with paired baseline and D100 post-ASCT samples

## Slide 5
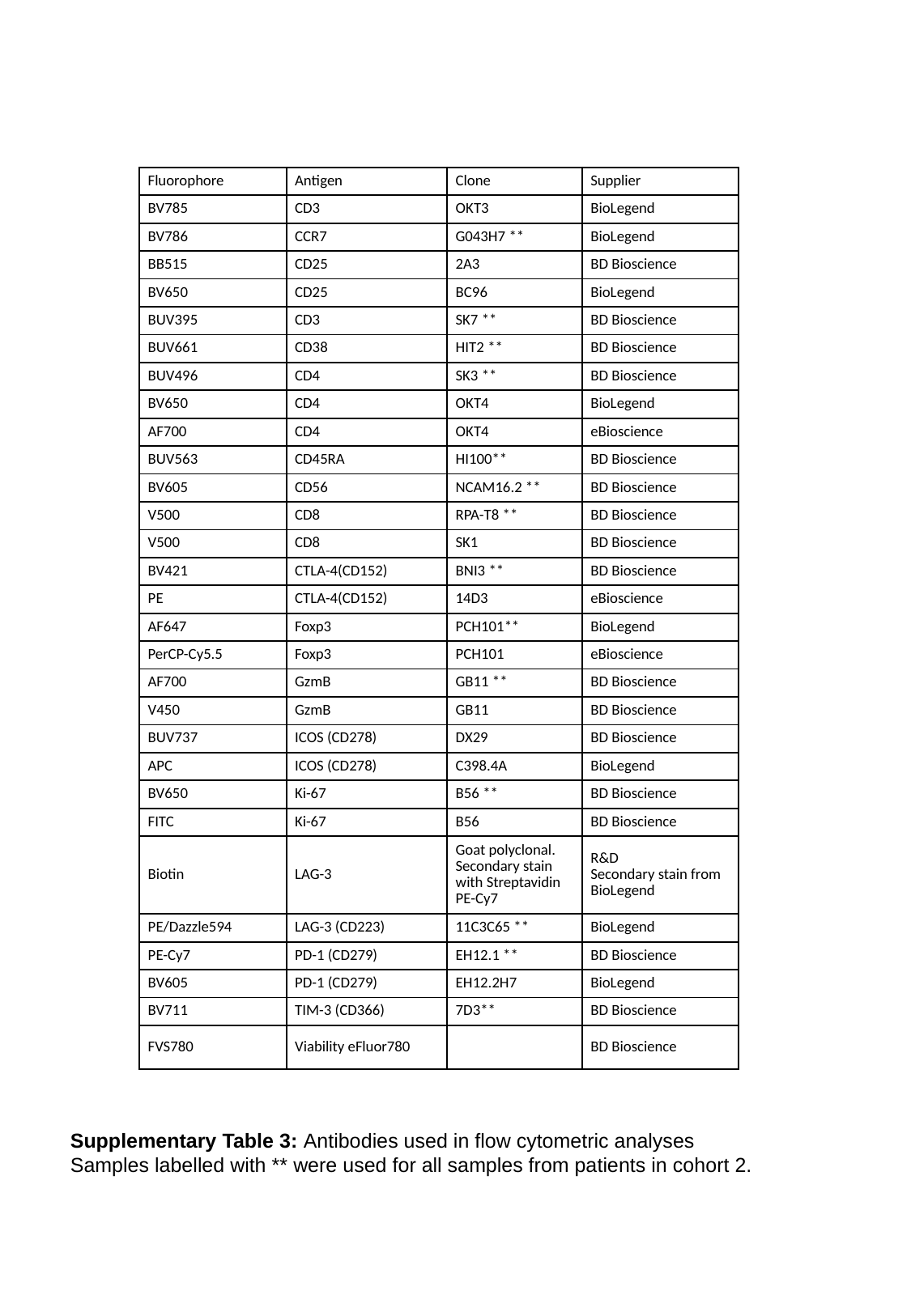

| Fluorophore | Antigen | Clone | Supplier |
| --- | --- | --- | --- |
| BV785 | CD3 | OKT3 | BioLegend |
| BV786 | CCR7 | G043H7 \*\* | BioLegend |
| BB515 | CD25 | 2A3 | BD Bioscience |
| BV650 | CD25 | BC96 | BioLegend |
| BUV395 | CD3 | SK7 \*\* | BD Bioscience |
| BUV661 | CD38 | HIT2 \*\* | BD Bioscience |
| BUV496 | CD4 | SK3 \*\* | BD Bioscience |
| BV650 | CD4 | OKT4 | BioLegend |
| AF700 | CD4 | OKT4 | eBioscience |
| BUV563 | CD45RA | HI100\*\* | BD Bioscience |
| BV605 | CD56 | NCAM16.2 \*\* | BD Bioscience |
| V500 | CD8 | RPA-T8 \*\* | BD Bioscience |
| V500 | CD8 | SK1 | BD Bioscience |
| BV421 | CTLA-4(CD152) | BNI3 \*\* | BD Bioscience |
| PE | CTLA-4(CD152) | 14D3 | eBioscience |
| AF647 | Foxp3 | PCH101\*\* | BioLegend |
| PerCP-Cy5.5 | Foxp3 | PCH101 | eBioscience |
| AF700 | GzmB | GB11 \*\* | BD Bioscience |
| V450 | GzmB | GB11 | BD Bioscience |
| BUV737 | ICOS (CD278) | DX29 | BD Bioscience |
| APC | ICOS (CD278) | C398.4A | BioLegend |
| BV650 | Ki-67 | B56 \*\* | BD Bioscience |
| FITC | Ki-67 | B56 | BD Bioscience |
| Biotin | LAG-3 | Goat polyclonal. Secondary stain with Streptavidin PE-Cy7 | R&D Secondary stain from BioLegend |
| PE/Dazzle594 | LAG-3 (CD223) | 11C3C65 \*\* | BioLegend |
| PE-Cy7 | PD-1 (CD279) | EH12.1 \*\* | BD Bioscience |
| BV605 | PD-1 (CD279) | EH12.2H7 | BioLegend |
| BV711 | TIM-3 (CD366) | 7D3\*\* | BD Bioscience |
| FVS780 | Viability eFluor780 | | BD Bioscience |
Supplementary Table 3: Antibodies used in flow cytometric analyses
Samples labelled with ** were used for all samples from patients in cohort 2.

## Slide 6
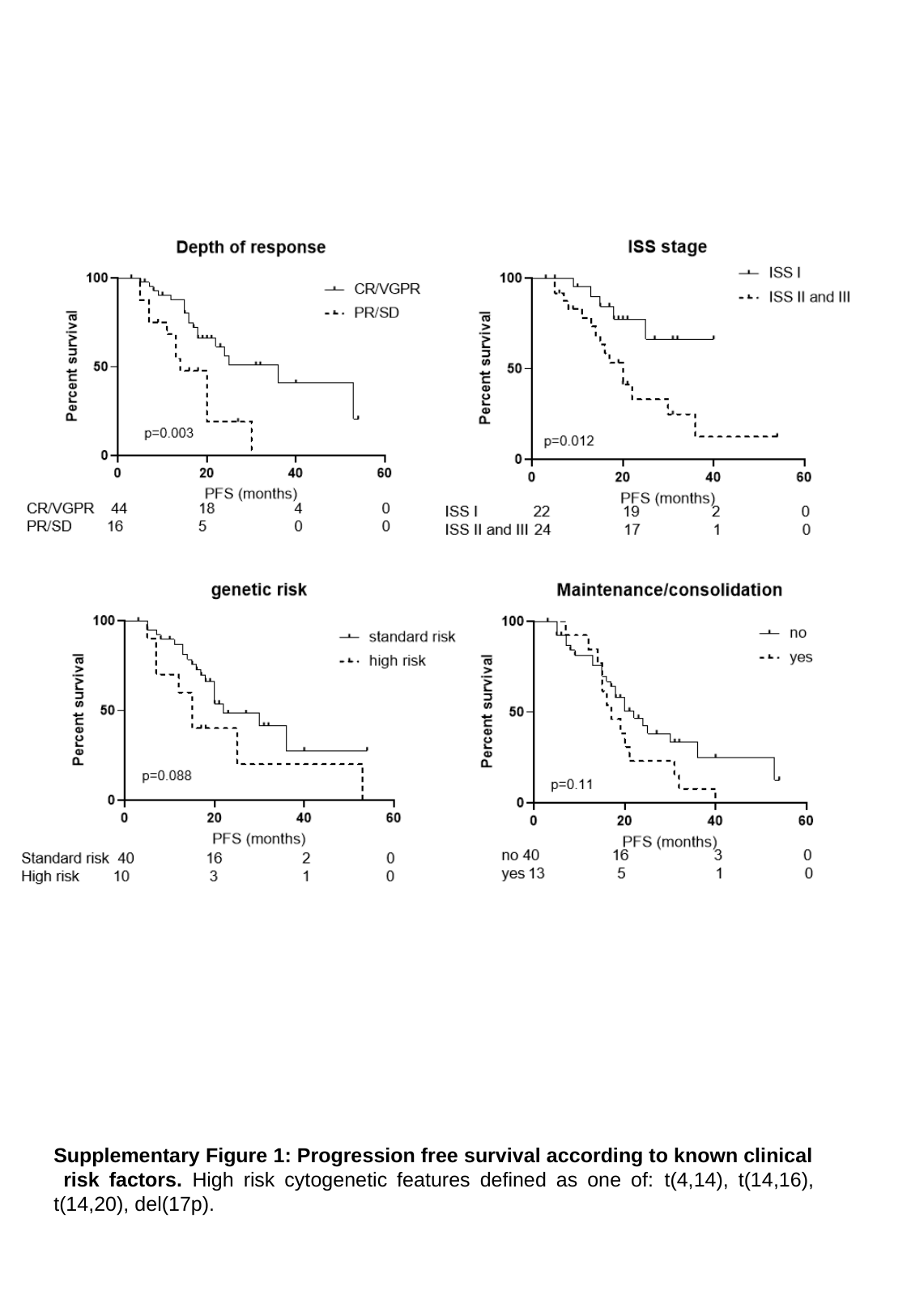

Supplementary Figure 1: Progression free survival according to known clinical risk factors. High risk cytogenetic features defined as one of: t(4,14), t(14,16), t(14,20), del(17p).

## Slide 7
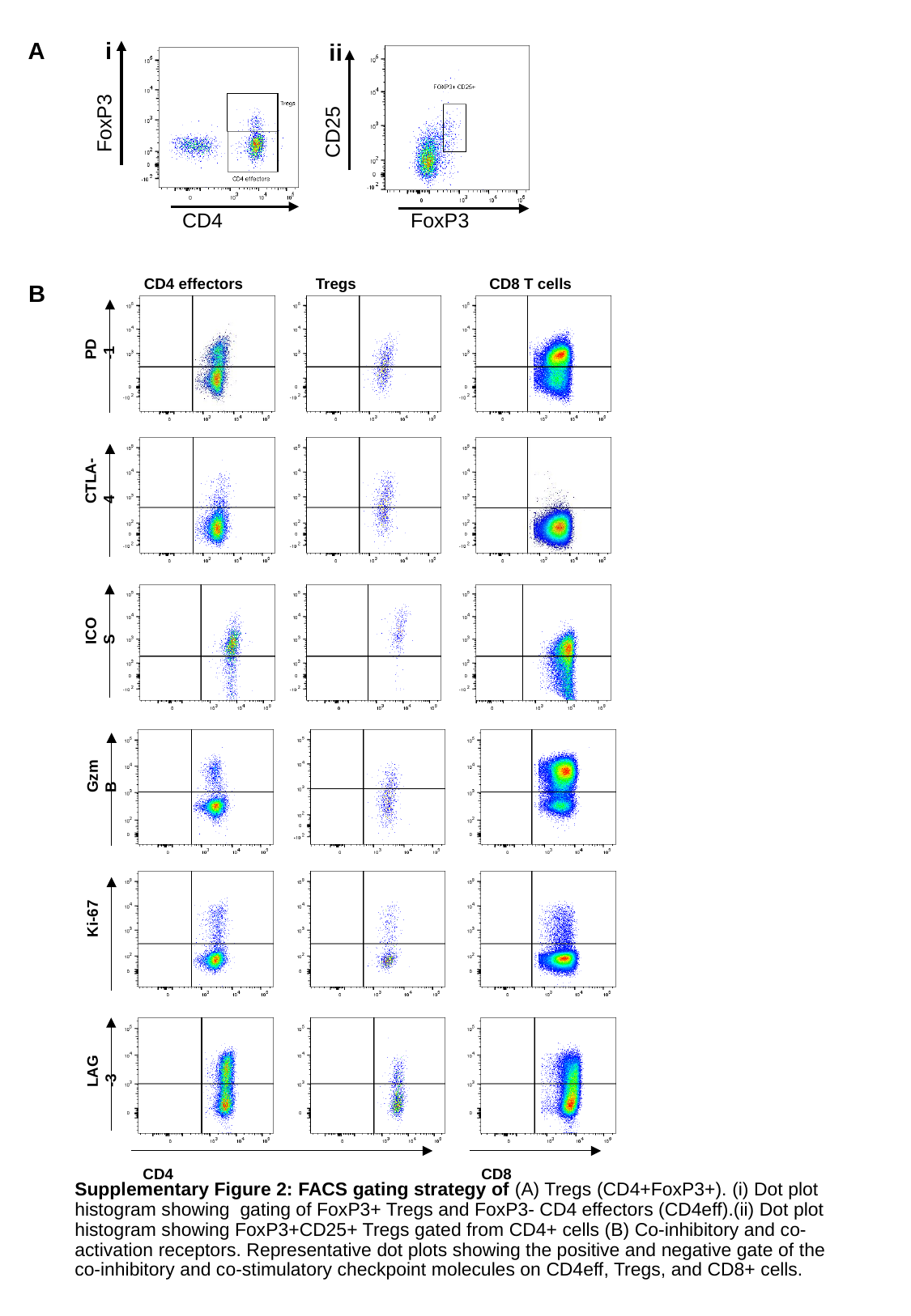

i
A
ii
FoxP3
CD25
FoxP3
CD4
CD4 effectors
Tregs
CD8 T cells
B
PD-1
CTLA-4
ICOS
GzmB
Ki-67
LAG-3
CD4
CD8
Supplementary Figure 2: FACS gating strategy of (A) Tregs (CD4+FoxP3+). (i) Dot plot histogram showing gating of FoxP3+ Tregs and FoxP3- CD4 effectors (CD4eff).(ii) Dot plot histogram showing FoxP3+CD25+ Tregs gated from CD4+ cells (B) Co-inhibitory and co-activation receptors. Representative dot plots showing the positive and negative gate of the co-inhibitory and co-stimulatory checkpoint molecules on CD4eff, Tregs, and CD8+ cells.

## Slide 8
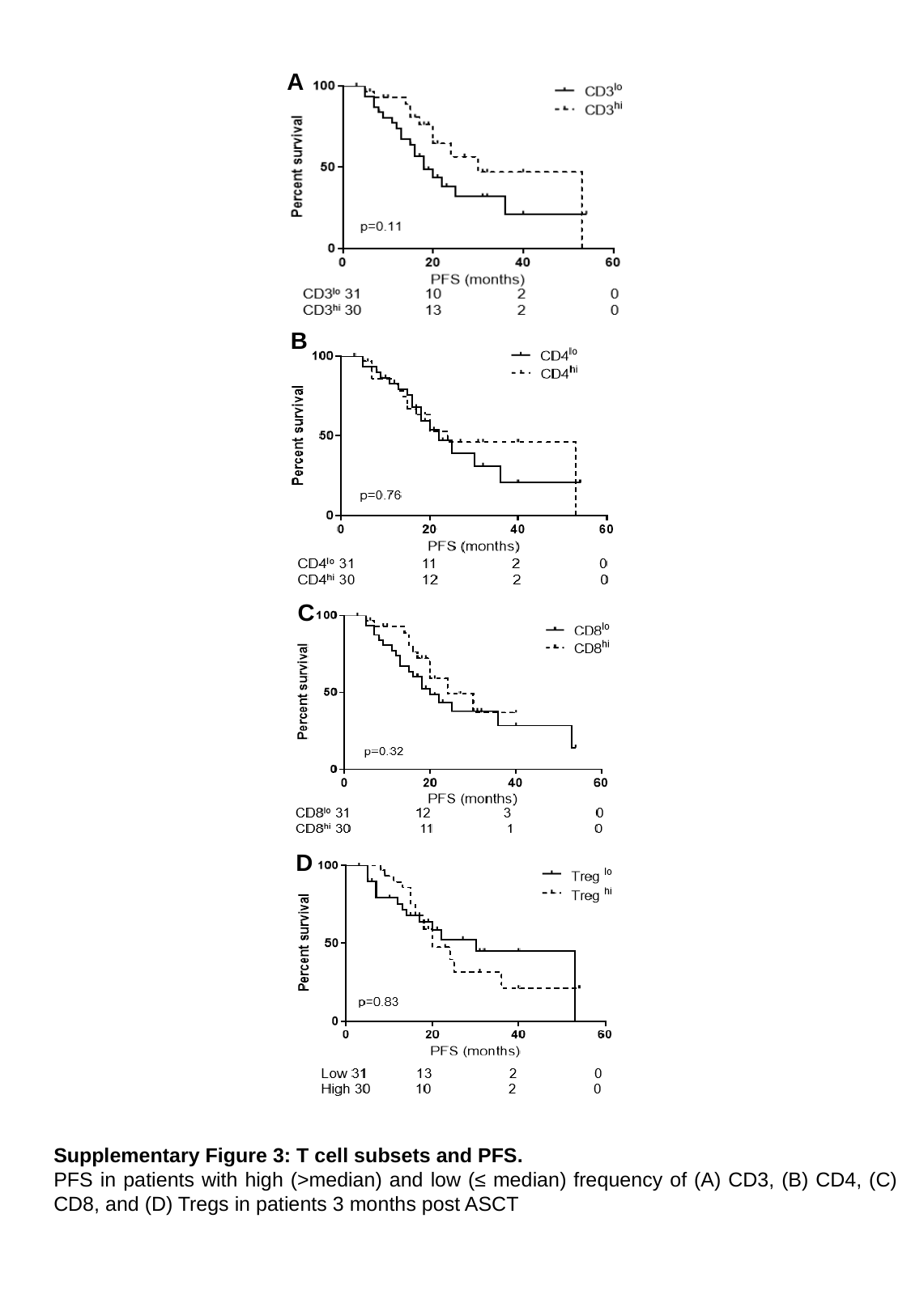

A
B
C
D
Supplementary Figure 3: T cell subsets and PFS.
PFS in patients with high (>median) and low (≤ median) frequency of (A) CD3, (B) CD4, (C) CD8, and (D) Tregs in patients 3 months post ASCT

## Slide 9
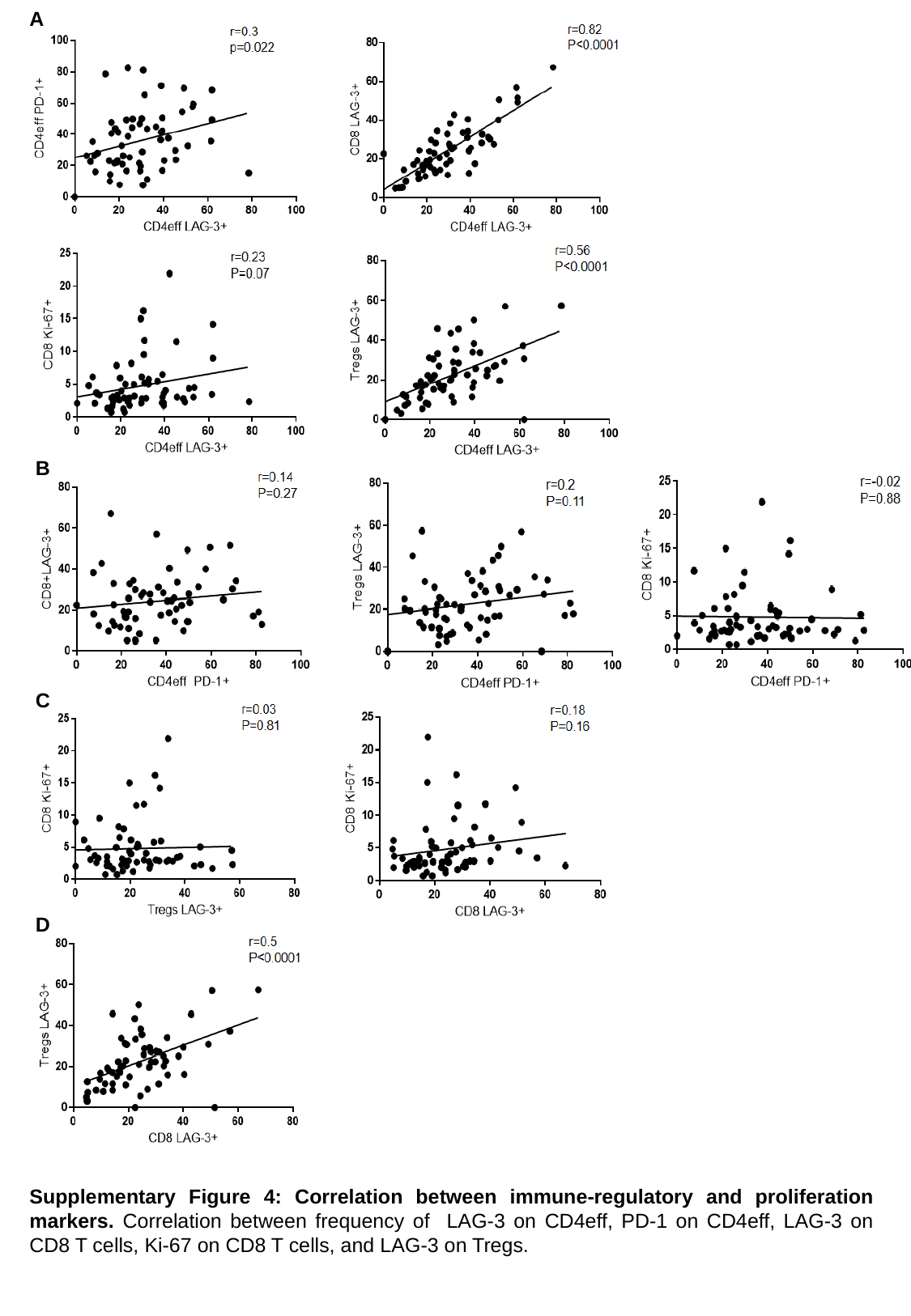

A
B
C
D
Supplementary Figure 4: Correlation between immune-regulatory and proliferation markers. Correlation between frequency of LAG-3 on CD4eff, PD-1 on CD4eff, LAG-3 on CD8 T cells, Ki-67 on CD8 T cells, and LAG-3 on Tregs.

## Slide 10
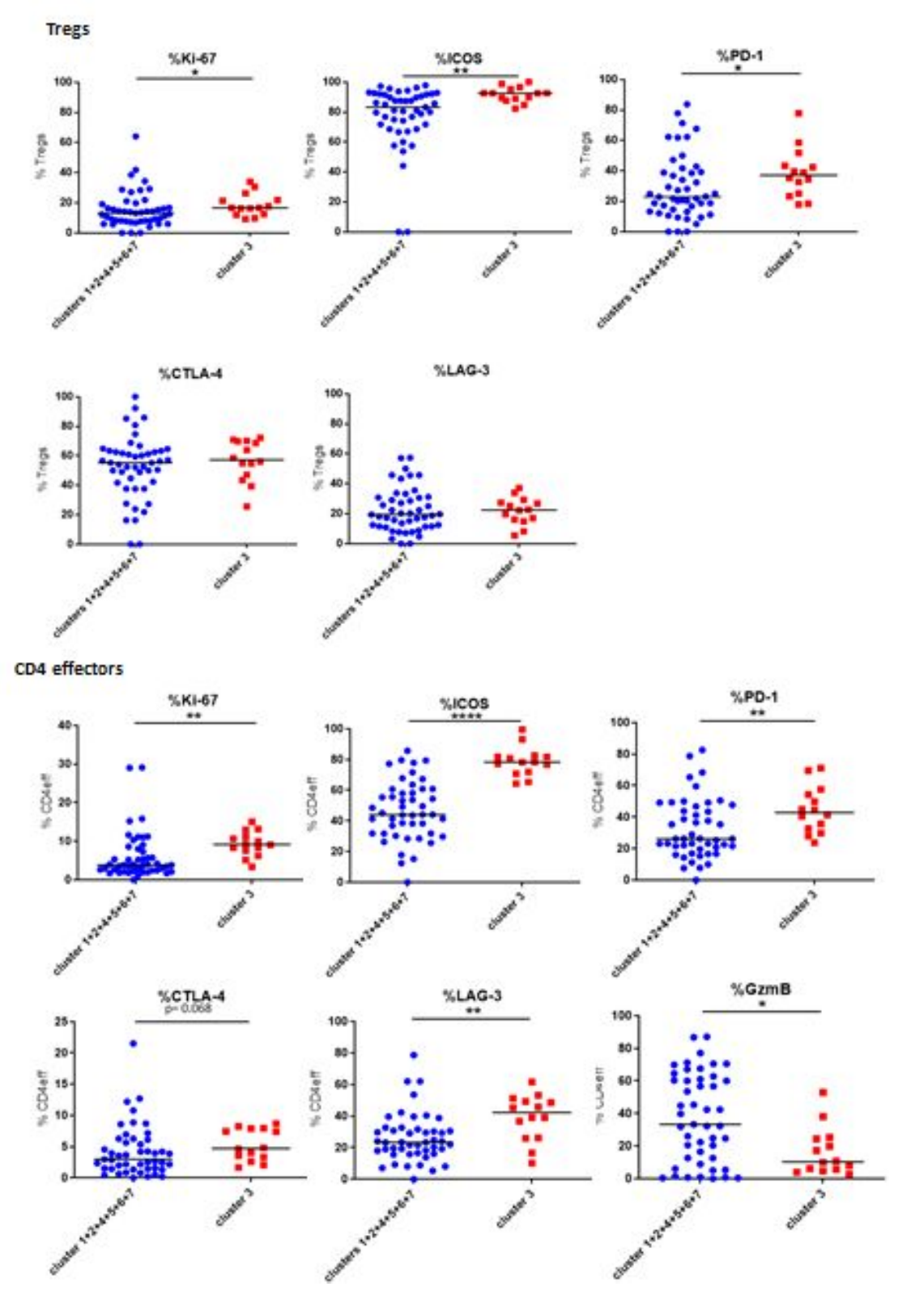

## Slide 11
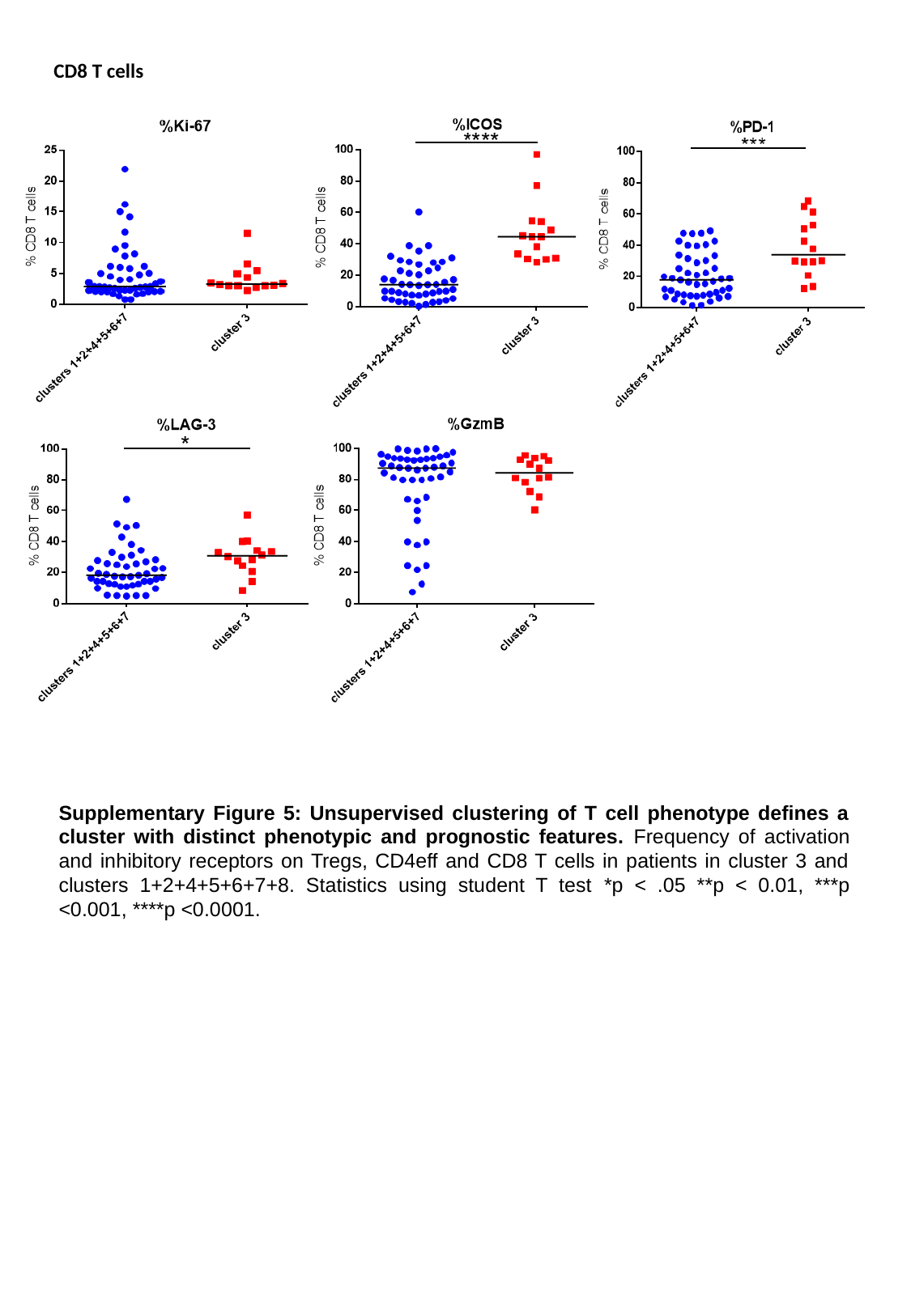

CD8 T cells
Supplementary Figure 5: Unsupervised clustering of T cell phenotype defines a cluster with distinct phenotypic and prognostic features. Frequency of activation and inhibitory receptors on Tregs, CD4eff and CD8 T cells in patients in cluster 3 and clusters 1+2+4+5+6+7+8. Statistics using student T test *p < .05 **p < 0.01, ***p <0.001, ****p <0.0001.

## Slide 12
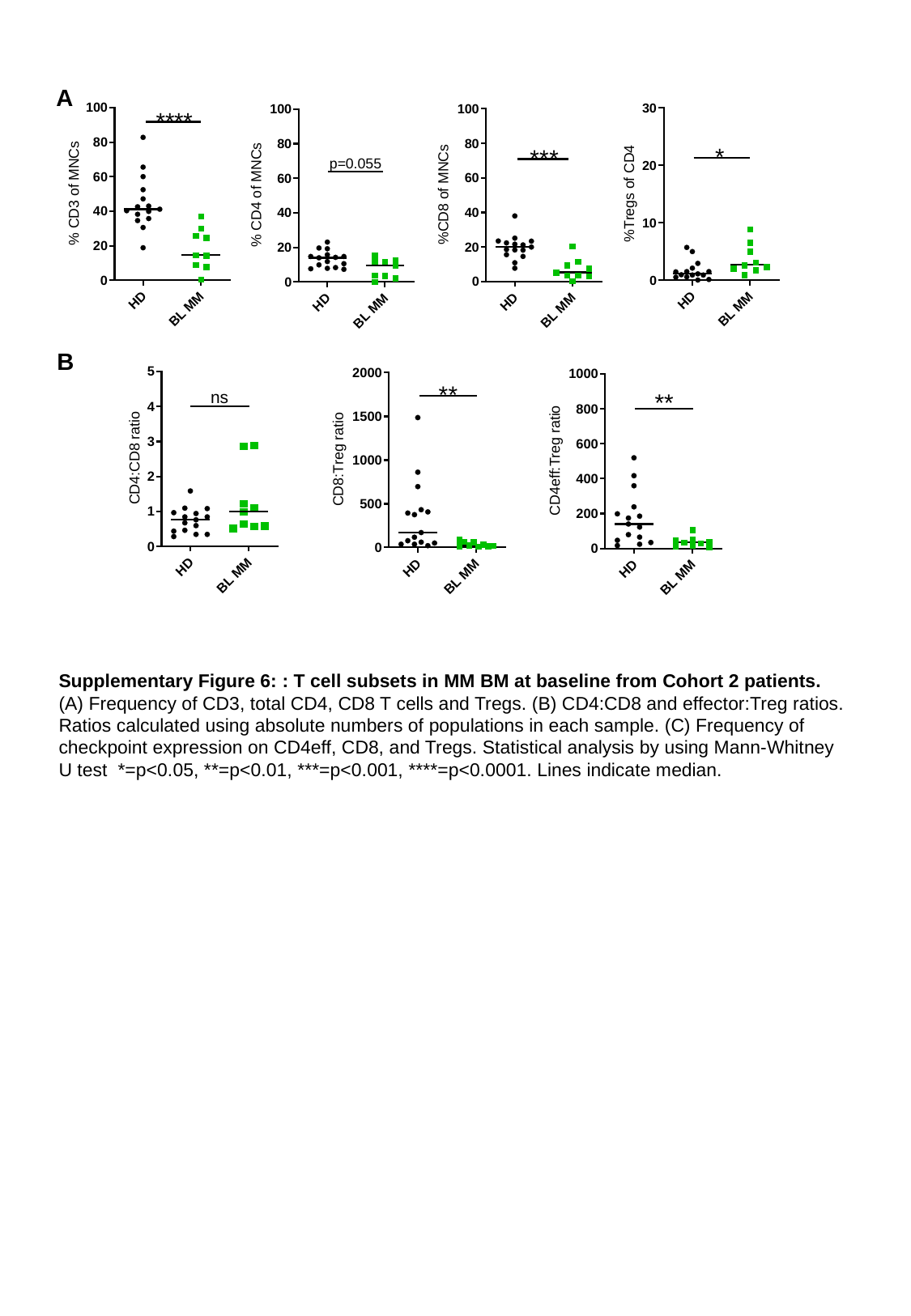

A
B
Supplementary Figure 6: : T cell subsets in MM BM at baseline from Cohort 2 patients. (A) Frequency of CD3, total CD4, CD8 T cells and Tregs. (B) CD4:CD8 and effector:Treg ratios. Ratios calculated using absolute numbers of populations in each sample. (C) Frequency of checkpoint expression on CD4eff, CD8, and Tregs. Statistical analysis by using Mann-Whitney U test *=p<0.05, **=p<0.01, ***=p<0.001, ****=p<0.0001. Lines indicate median.

## Slide 13
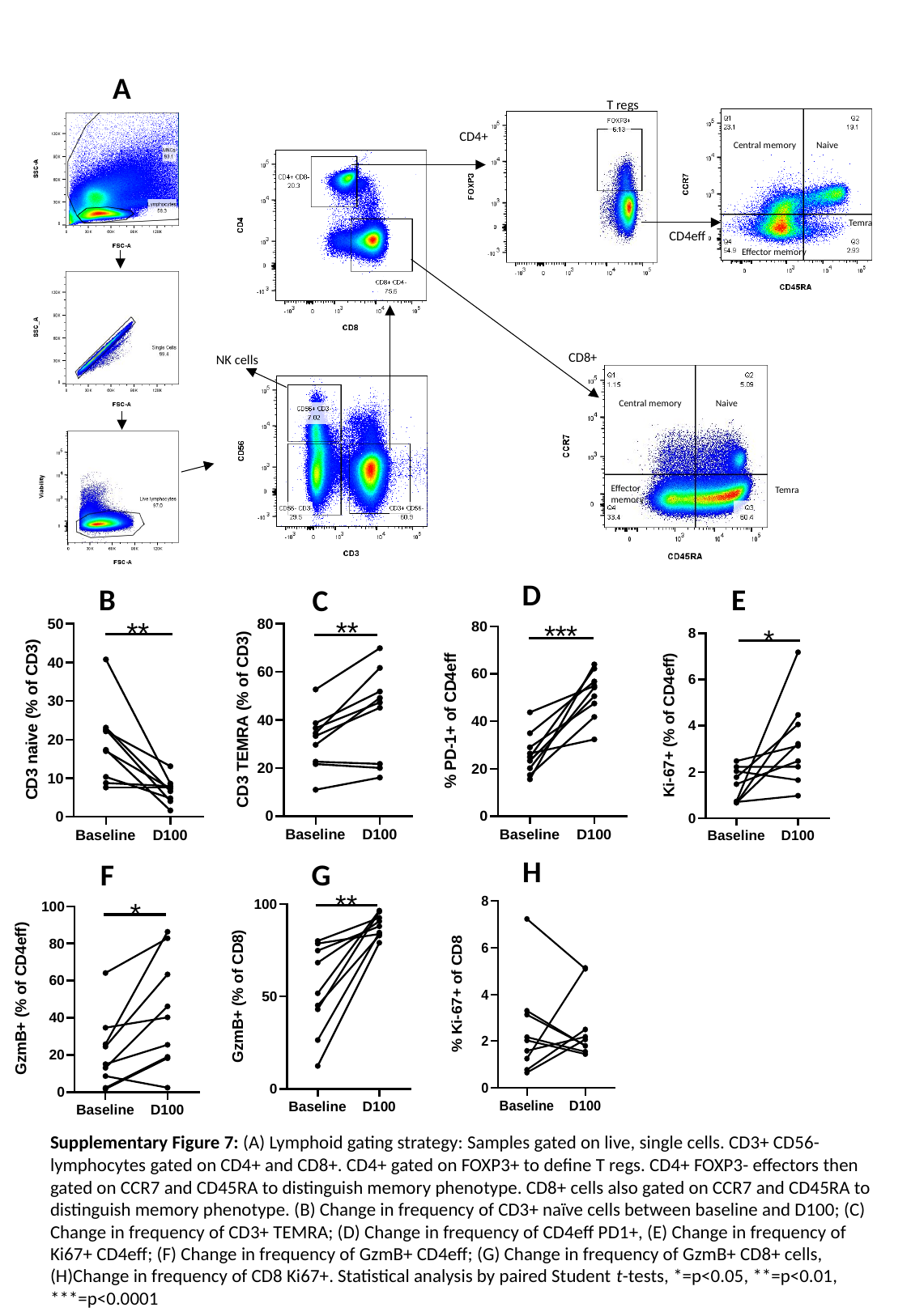

A
T regs
CD4+
Central memory
Naive
Temra
CD4eff
Effector memory
CD8+
NK cells
Naive
Central memory
Effector memory
Temra
D
B
E
C
H
F
G
Supplementary Figure 7: (A) Lymphoid gating strategy: Samples gated on live, single cells. CD3+ CD56- lymphocytes gated on CD4+ and CD8+. CD4+ gated on FOXP3+ to define T regs. CD4+ FOXP3- effectors then gated on CCR7 and CD45RA to distinguish memory phenotype. CD8+ cells also gated on CCR7 and CD45RA to distinguish memory phenotype. (B) Change in frequency of CD3+ naïve cells between baseline and D100; (C) Change in frequency of CD3+ TEMRA; (D) Change in frequency of CD4eff PD1+, (E) Change in frequency of Ki67+ CD4eff; (F) Change in frequency of GzmB+ CD4eff; (G) Change in frequency of GzmB+ CD8+ cells, (H)Change in frequency of CD8 Ki67+. Statistical analysis by paired Student t-tests, *=p<0.05, **=p<0.01, ***=p<0.0001

## Slide 14
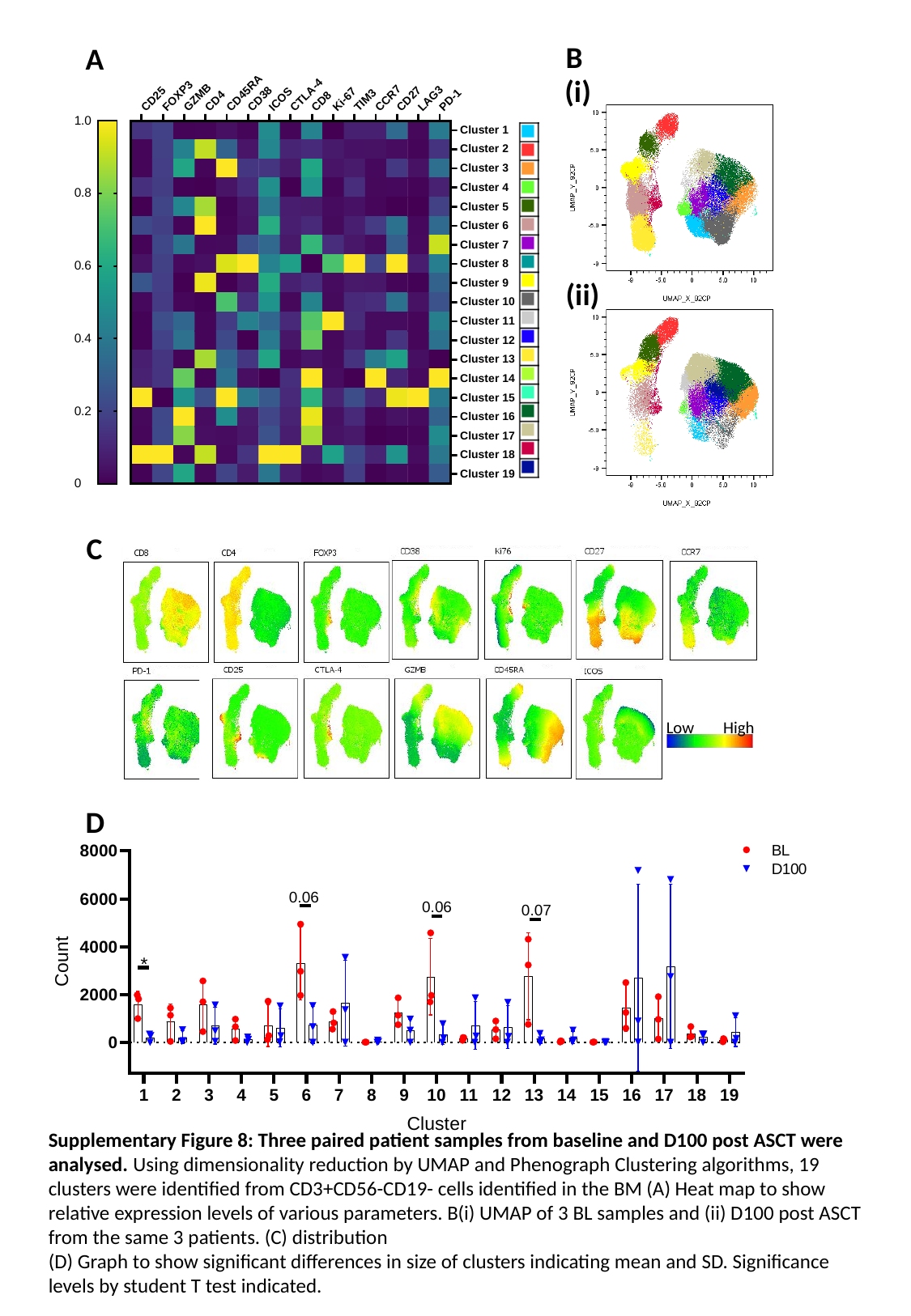

B
A
(i)
(ii)
C
Low
High
D
Supplementary Figure 8: Three paired patient samples from baseline and D100 post ASCT were analysed. Using dimensionality reduction by UMAP and Phenograph Clustering algorithms, 19 clusters were identified from CD3+CD56-CD19- cells identified in the BM (A) Heat map to show relative expression levels of various parameters. B(i) UMAP of 3 BL samples and (ii) D100 post ASCT from the same 3 patients. (C) distribution
(D) Graph to show significant differences in size of clusters indicating mean and SD. Significance levels by student T test indicated.

## Slide 15
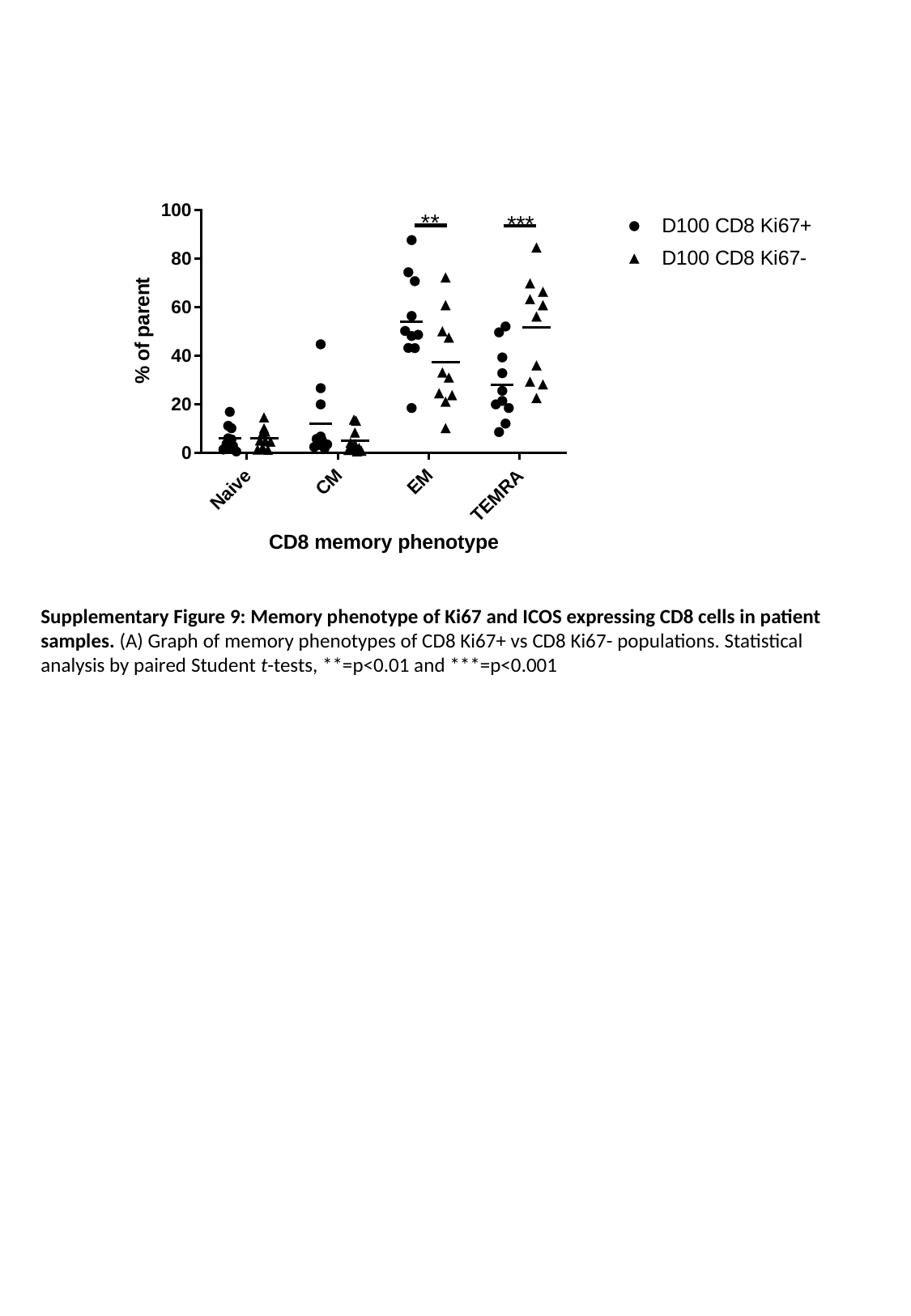

Supplementary Figure 9: Memory phenotype of Ki67 and ICOS expressing CD8 cells in patient samples. (A) Graph of memory phenotypes of CD8 Ki67+ vs CD8 Ki67- populations. Statistical analysis by paired Student t-tests, **=p<0.01 and ***=p<0.001

## Slide 16
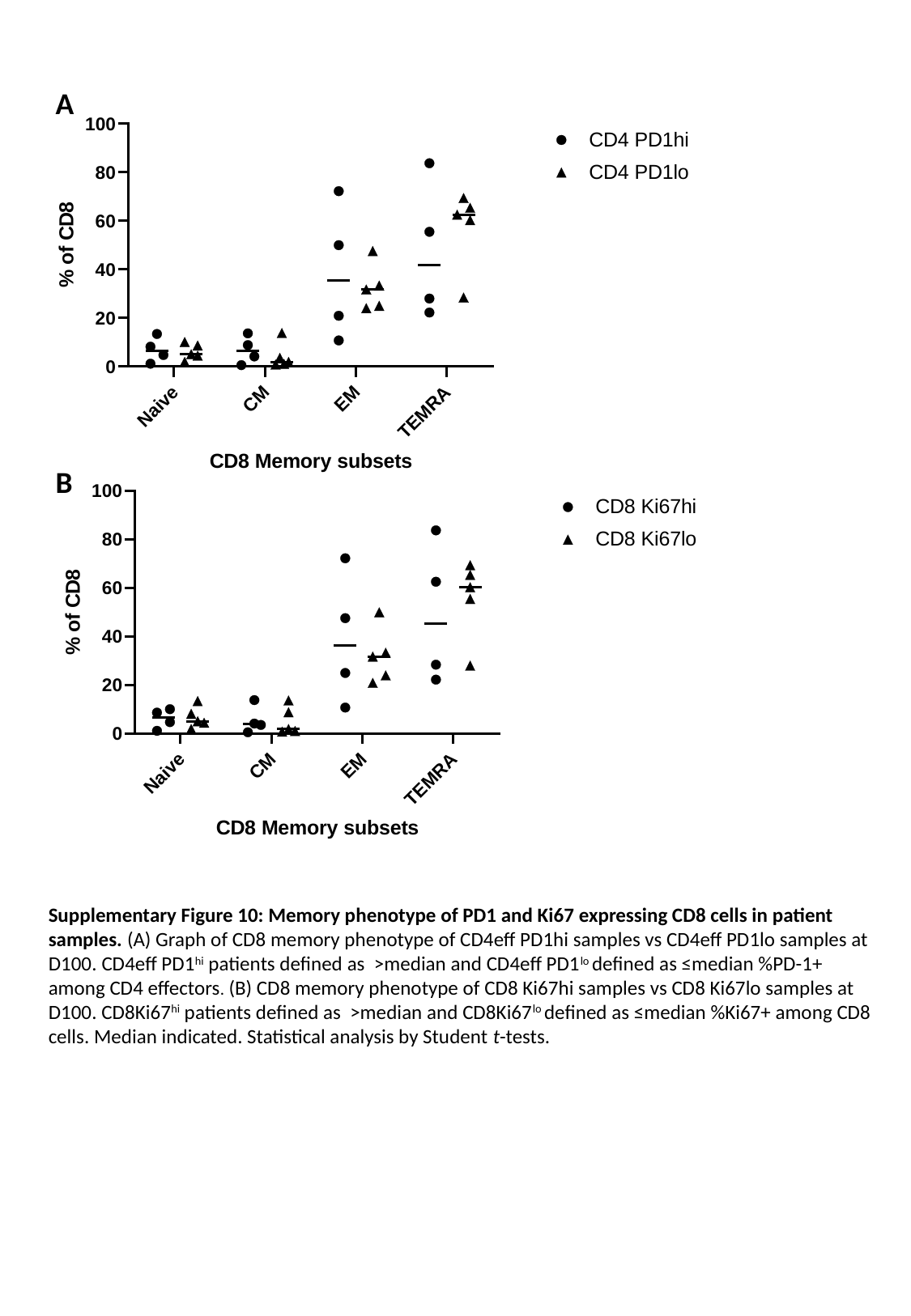

A
B
Supplementary Figure 10: Memory phenotype of PD1 and Ki67 expressing CD8 cells in patient samples. (A) Graph of CD8 memory phenotype of CD4eff PD1hi samples vs CD4eff PD1lo samples at D100. CD4eff PD1hi patients defined as >median and CD4eff PD1lo defined as ≤median %PD-1+ among CD4 effectors. (B) CD8 memory phenotype of CD8 Ki67hi samples vs CD8 Ki67lo samples at D100. CD8Ki67hi patients defined as >median and CD8Ki67lo defined as ≤median %Ki67+ among CD8 cells. Median indicated. Statistical analysis by Student t-tests.

## Slide 17
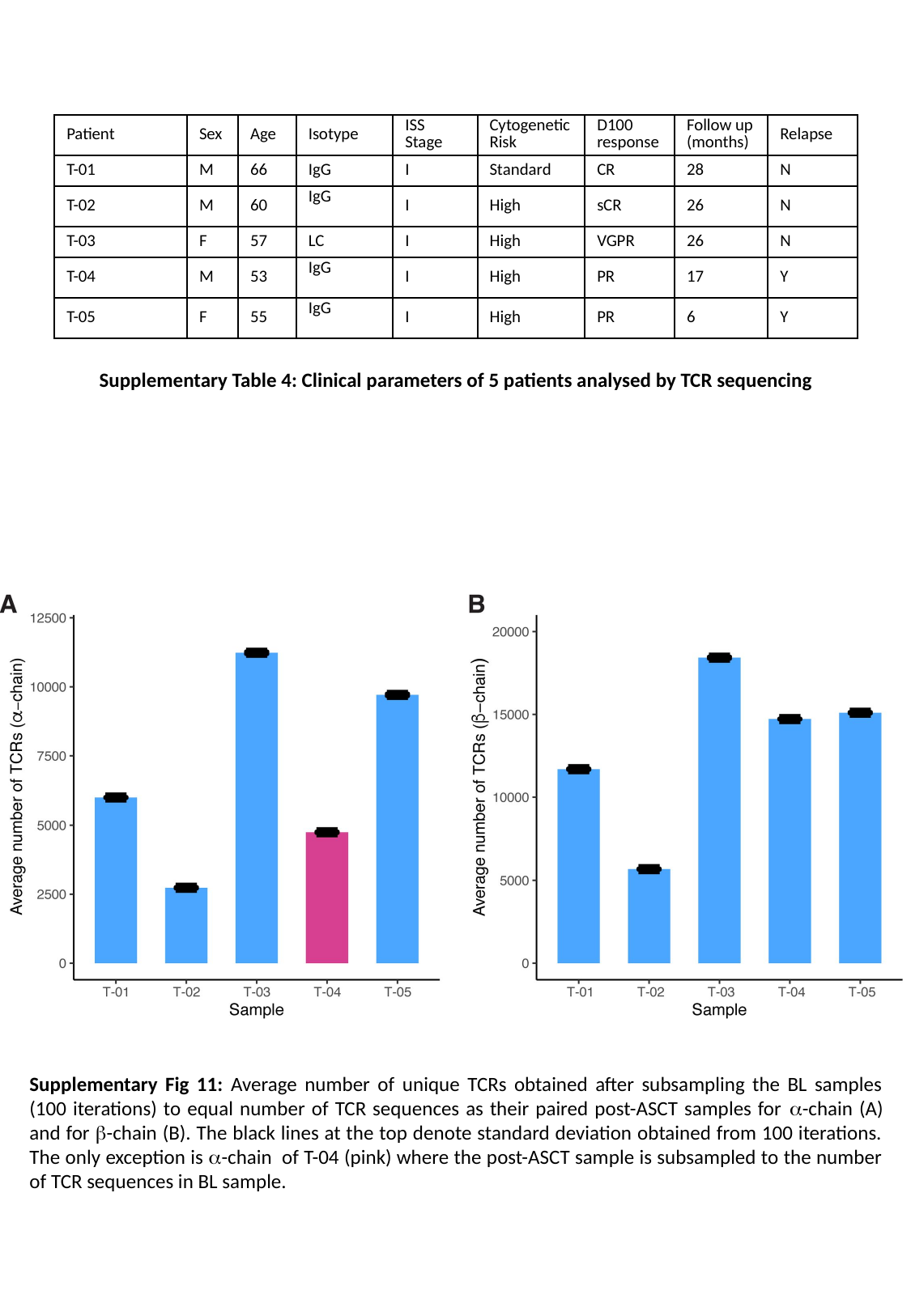

| Patient | Sex | Age | Isotype | ISS Stage | Cytogenetic Risk | D100 response | Follow up (months) | Relapse |
| --- | --- | --- | --- | --- | --- | --- | --- | --- |
| T-01 | M | 66 | IgG | I | Standard | CR | 28 | N |
| T-02 | M | 60 | IgG | I | High | sCR | 26 | N |
| T-03 | F | 57 | LC | I | High | VGPR | 26 | N |
| T-04 | M | 53 | IgG | I | High | PR | 17 | Y |
| T-05 | F | 55 | IgG | I | High | PR | 6 | Y |
Supplementary Table 4: Clinical parameters of 5 patients analysed by TCR sequencing
Supplementary Fig 11: Average number of unique TCRs obtained after subsampling the BL samples (100 iterations) to equal number of TCR sequences as their paired post-ASCT samples for α-chain (A) and for β-chain (B). The black lines at the top denote standard deviation obtained from 100 iterations. The only exception is α-chain  of T-04 (pink) where the post-ASCT sample is subsampled to the number of TCR sequences in BL sample.

## Slide 18
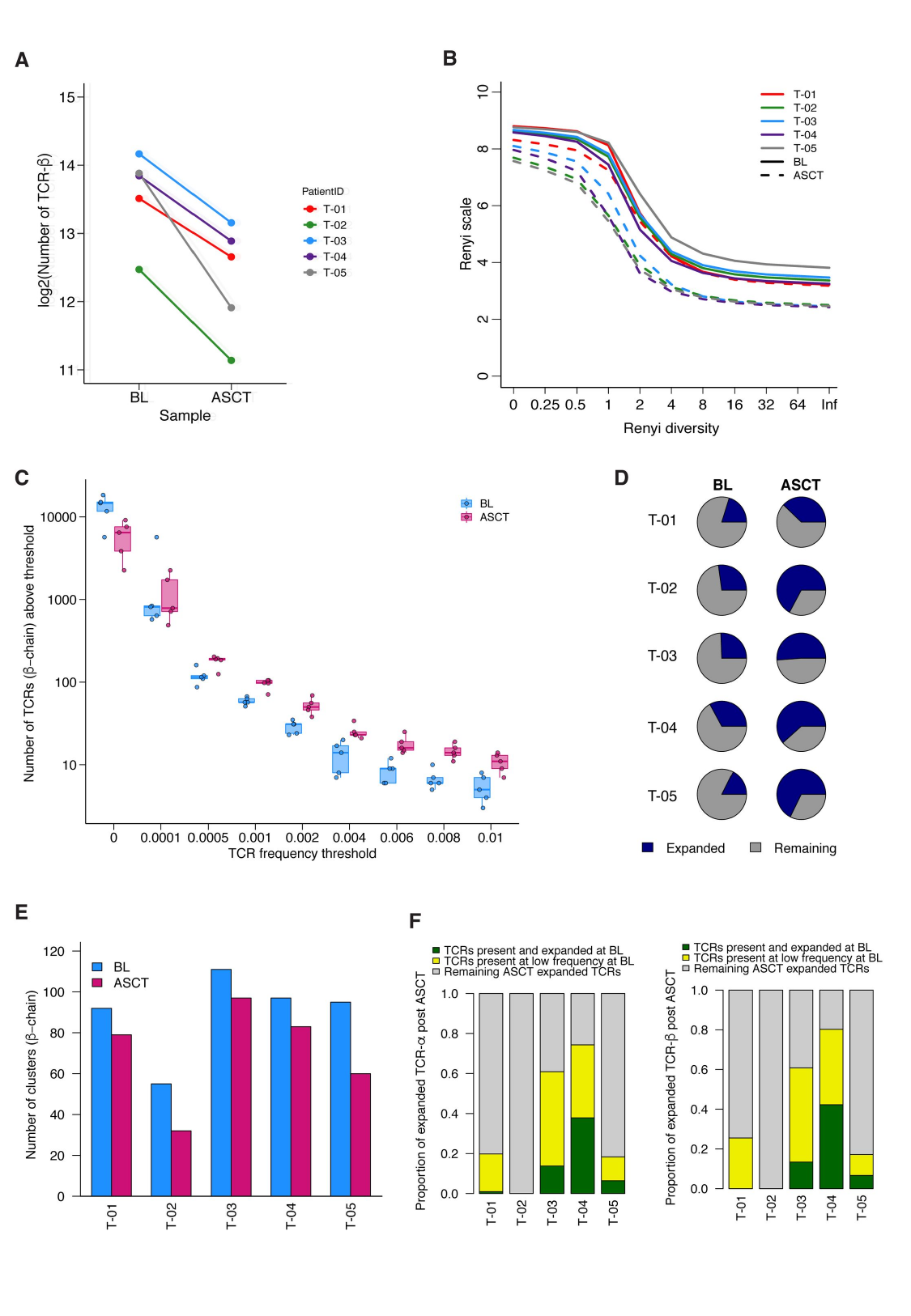

## Slide 19
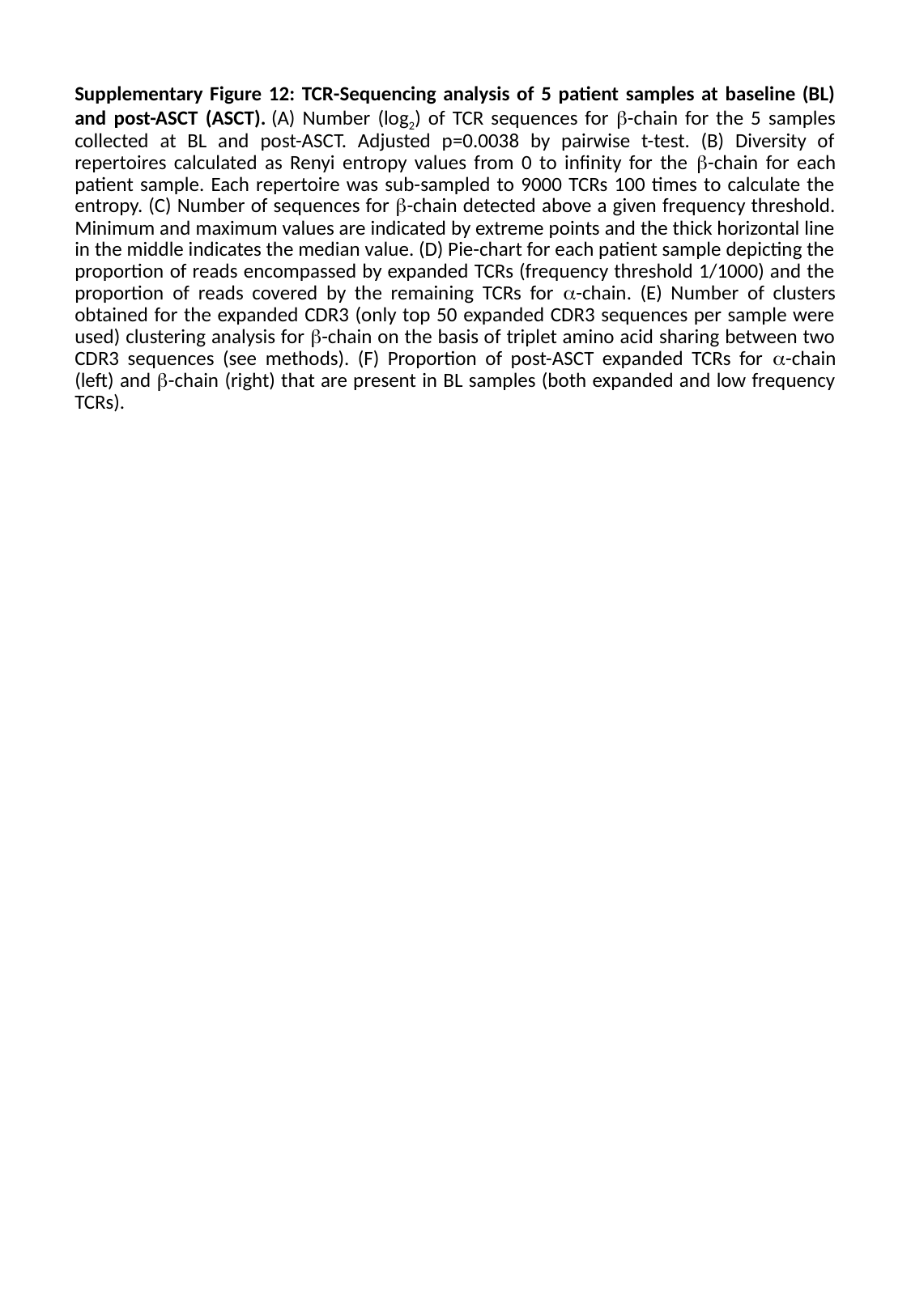

Supplementary Figure 12: TCR-Sequencing analysis of 5 patient samples at baseline (BL) and post-ASCT (ASCT). (A) Number (log2) of TCR sequences for β-chain for the 5 samples collected at BL and post-ASCT. Adjusted p=0.0038 by pairwise t-test. (B) Diversity of repertoires calculated as Renyi entropy values from 0 to infinity for the β-chain for each patient sample. Each repertoire was sub-sampled to 9000 TCRs 100 times to calculate the entropy. (C) Number of sequences for β-chain detected above a given frequency threshold. Minimum and maximum values are indicated by extreme points and the thick horizontal line in the middle indicates the median value. (D) Pie-chart for each patient sample depicting the proportion of reads encompassed by expanded TCRs (frequency threshold 1/1000) and the proportion of reads covered by the remaining TCRs for α-chain. (E) Number of clusters obtained for the expanded CDR3 (only top 50 expanded CDR3 sequences per sample were used) clustering analysis for β-chain on the basis of triplet amino acid sharing between two CDR3 sequences (see methods). (F) Proportion of post-ASCT expanded TCRs for α-chain (left) and β-chain (right) that are present in BL samples (both expanded and low frequency TCRs).

## Slide 20
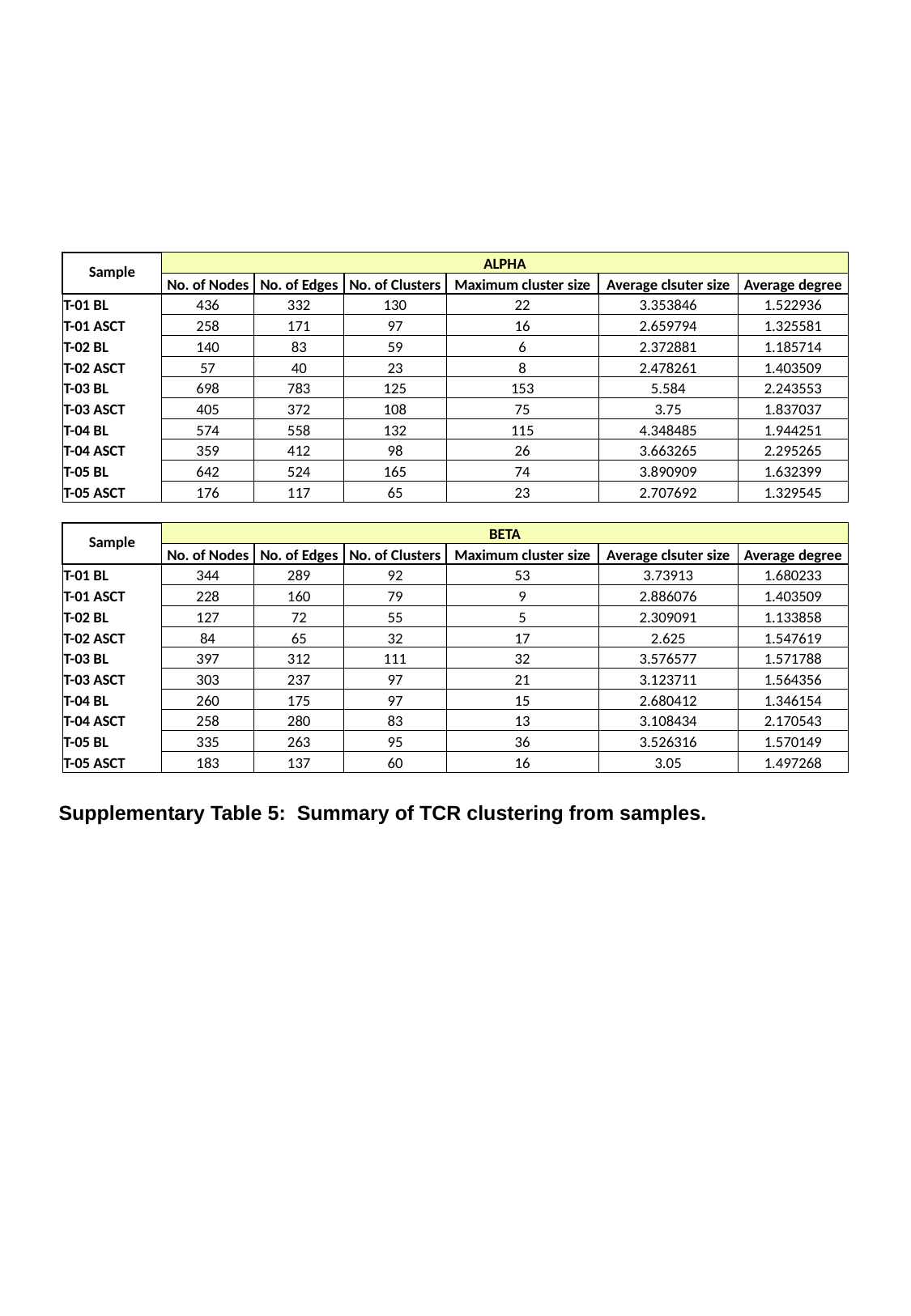

| Sample | ALPHA | | | | | |
| --- | --- | --- | --- | --- | --- | --- |
| | No. of Nodes | No. of Edges | No. of Clusters | Maximum cluster size | Average clsuter size | Average degree |
| T-01 BL | 436 | 332 | 130 | 22 | 3.353846 | 1.522936 |
| T-01 ASCT | 258 | 171 | 97 | 16 | 2.659794 | 1.325581 |
| T-02 BL | 140 | 83 | 59 | 6 | 2.372881 | 1.185714 |
| T-02 ASCT | 57 | 40 | 23 | 8 | 2.478261 | 1.403509 |
| T-03 BL | 698 | 783 | 125 | 153 | 5.584 | 2.243553 |
| T-03 ASCT | 405 | 372 | 108 | 75 | 3.75 | 1.837037 |
| T-04 BL | 574 | 558 | 132 | 115 | 4.348485 | 1.944251 |
| T-04 ASCT | 359 | 412 | 98 | 26 | 3.663265 | 2.295265 |
| T-05 BL | 642 | 524 | 165 | 74 | 3.890909 | 1.632399 |
| T-05 ASCT | 176 | 117 | 65 | 23 | 2.707692 | 1.329545 |
| | | | | | | |
| Sample | BETA | | | | | |
| | No. of Nodes | No. of Edges | No. of Clusters | Maximum cluster size | Average clsuter size | Average degree |
| T-01 BL | 344 | 289 | 92 | 53 | 3.73913 | 1.680233 |
| T-01 ASCT | 228 | 160 | 79 | 9 | 2.886076 | 1.403509 |
| T-02 BL | 127 | 72 | 55 | 5 | 2.309091 | 1.133858 |
| T-02 ASCT | 84 | 65 | 32 | 17 | 2.625 | 1.547619 |
| T-03 BL | 397 | 312 | 111 | 32 | 3.576577 | 1.571788 |
| T-03 ASCT | 303 | 237 | 97 | 21 | 3.123711 | 1.564356 |
| T-04 BL | 260 | 175 | 97 | 15 | 2.680412 | 1.346154 |
| T-04 ASCT | 258 | 280 | 83 | 13 | 3.108434 | 2.170543 |
| T-05 BL | 335 | 263 | 95 | 36 | 3.526316 | 1.570149 |
| T-05 ASCT | 183 | 137 | 60 | 16 | 3.05 | 1.497268 |
Supplementary Table 5: Summary of TCR clustering from samples.
